# BRI1 and BAK1 Canonical Distribution in Plasma Membrane Is HSP90 Dependent

**DOI:** 10.3390/cells11213341

**Published:** 2022-10-22

**Authors:** Despina Samakovli, Loukia Roka, Panagiota Konstantinia Plitsi, Georgia Drakakaki, Kosmas Haralampidis, Dimitrios J. Stravopodis, Polydefkis Hatzopoulos, Dimitra Milioni

**Affiliations:** 1 Biotechnology Department, Agricultural University of Athens, Iera Odos 75, 11855 Athens, Greece; 2 Department of Plant Sciences, University of California, Davis, CA 95616, USA; 3 Biology Department, National and Kapodistrian University of Athens, 15701 Athens, Greece

**Keywords:** hormone, response, development, stress, HSP90, brassinosteroid, signaling, *Arabidopsis*, receptor, membrane

## Abstract

The activation of BRASSINOSTEROID INSENSITIVE1 (BRI1) and its association with the BRI1 ASSOCIATED RECEPTOR KINASE1 (BAK1) are key steps for the initiation of the BR signaling cascade mediating hypocotyl elongation. Heat shock protein 90 (HSP90) is crucial in the regulation of signaling processes and the activation of hormonal receptors. We report that HSP90 is required for the maintenance of the BRI1 receptor at the plasma membrane (PM) and its association with the BAK1 co-receptor during BL-ligand stimulation. HSP90 mediates BR perception and signal transduction through physical interactions with BRI1 and BAK1, while chaperone depletion resulted in lower levels of BRI1 and BAK1 receptors at the PM and affected the spatial partitioning and organization of BRI1/BAK1 heterocomplexes at the PM. The BRI1/BAK1 interaction relies on the HSP90-dependent activation of the kinase domain of BRI1 which leads to the confinement of the spatial dynamics of the membrane resident BRI1 and the attenuation of the downstream signaling. This is evident by the impaired activation and transcriptional activity of BRI1 EMS SUPPRESSOR 1 (BES1) upon HSP90 depletion. Our findings provide conclusive evidence that further expands the commitment of HSP90 in BR signaling through the HSP90-mediated activation of BRI1 in the control of the BR signaling cascade in plants.

## 1. Introduction

In plant development, the integration of environmental and internal cues to control growth relies on signal perception processes. It is largely unknown how diverse signals affect plasma membrane (PM) receptors to sustain activated conformation and facilitate intracellular communication, environmental sensing, and directional growth. Multimerization and post-translational modifications are a few examples of the receptor dynamic cycle in signal perception-transduction pathways [1], while endocytosis, recycling, and degradation modulate the localization, abundance, and activity of receptors at the PM [2].

Plant steroids, such as brassinosteroids (BR), act synergistically with other hormones to promote development from the early stages of embryogenesis to flower pattern formation [3,4]. The extracellular domain of the PM receptor kinase BRI1 (BRASSINOSTEROID INSENSITIVE 1) binds to BR leading to its homodimerization. Activated BRI1 binds to BRI1 ASSOCIATED RECEPTOR KINASE1 (BAK1) and forms an active BR receptor complex that mediates plant steroid signaling [3,5]. The downstream signaling pathway results in the activation of BRI1 EMS SUPPRESSOR 1 (BES1) and BRASSINAZOLE RESISTANT 1 (BZR1), two key transcription factors that control the BR-responsive gene expression. Other components of BR signaling, such as BRASSINOSTEROID INSENSITIVE 2 (BIN2) and *bri1* SUPPRESSOR 1 (BSU1) serve as control points of the pathway [4]. The PM pool of BRI1 regulates BR signaling, while impaired BRI1 recycling through endocytosis results in constitutive BR responses [6,7,8,9]. It is debated whether BRI1 clathrin-mediated endocytosis is induced by ligand binding or whether BRI1 undergoes a ligand-induced endocytosis which relies on its ubiquitination [8,9,10,11,12].

Heat shock protein 90 (HSP90), a highly conserved molecular chaperone, acts as a regulatory hub in multiple biological networks as it is involved in the activation and stability of a variety of essential signaling components [13,14,15,16,17,18]. In addition, HSP90 is a key player in evolution providing insight into new functions in all studied organisms [14,19,20]. It is hypothesized that HSP90-mediated spatiotemporal compatibility and activity of its client proteins in signaling cascades, is the convergence of these two diverse features of HSP90 function.

There is a growing body of evidence that HSP90 plays a key role in hormonal signaling networks through interactions with components of auxin and jasmonate receptor complexes [18]. Furthermore, pharmacological and genetic evidence indicate that HSP90 impacts the signal transduction of abscisic acid (ABA) [18]. However, an HSP90-mediated crosstalk between plant hormones still remains elusive in plants. Previous studies have implicated HSP90 in the downstream signaling pathway of BR, as the activity of HSP90 sustained the BIN2 nuclear function [21] and modulated the BR-mediated feedback control of BES1 and BZR1 transcription factors [22,23,24,25,26]. HSP90 has also been shown to interact with the OsCERK1 chitin receptor, a PM receptor involved in rice innate immunity [27]. The role of HSP90 in the regulation of PM proteins was further supported by a recent study showing that the activity of HSP90 affects the PM pool by modulating the internalization of the PM-localized auxin transporter, PIN1 [28]. However, it is largely unknown whether the stability and activity of the BRI1 receptor at the PM during BR signaling require HSP90, as in the case of animal plasma membrane receptors such as ErbB-2 and HER2 [29].

In this study, we used complementary approaches to unravel the role of HSP90 at the onset of BR signaling. Specifically, this study aimed to address the following questions: (1) Do HSP90.1 and HSP90.2 physically interact with both BRI1 and BAK1 receptors? (2) How does genetic depletion of HSP90.1 or HSP90.2 modulate the abundance of BRI1 and BAK1 and alter the distribution of the receptors at the PM? (3) What are the mechanistic insights into the role of HSP90 in the BR activation and BRI1 heterodimerization with BAK1 co-receptor through the interactions of HSP90 with the LRR and kinase domains of BRI1 and BAK1, respectively? (4) How are the HSP90.1/BRI1 and BRI1/BAK1 complexes spatially distributed at the PM? Findings from this research will provide insight into the dynamics of the HSP90-dependent formation of BRI1 and BAK1 heterocomplexes during BR activation. It is plausible that the HSP90 constrains the PM dynamics of BRI1 and BAK1 receptors in heterogenous localities at the PM, attenuating BR signaling and BRI1-mediated hypocotyl elongation.

## 2. Materials and Method

### 2.1. Plant Growth Conditions

In the present study, *Arabidopsis thaliana* (L.) Heynh Columbia (Col-0) and WS-2 ecotypes were used as wild-type plants; mutant plants include *bri1-4* [30,31] *hsp90.1* (SALK_007614) and *hsp90.2* (SALK_038646). *Arabidopsis* wild-type ecotypes, mutants and transgenic, and *Nicotiana benthamiana* L. (tobacco) plants were grown under 16:8 h, light:dark cycles at 22 °C. *Arabidopsis* seeds were germinated on Murashige–Skoog (MS) medium. For hypocotyl length sensitivity assays wild-type and mutant plants were grown in the dark on vertical agar plates in plain MS medium or MS containing increasing HBL [22(S),23(S)-Homobrassinolide Sigma-Aldrich, St. Louis, MO, USA] concentrations (1–1000 nM) or 2 μΜ geldanamycin (GDA; Invivogen, San Diego, CA, USA).

### 2.2. Cloning Procedures and Fusion Constructs

The full length of the BAK1 co-receptor, BRI1 receptor, and BRI1LRR or BRI1 KS used for BiFC assays or Y2H assays was amplified by RT-PCR from RNA extracted from 7-d-old *Arabidopsis* seedlings using a specific set of primers (Supplementary Table S1). The fragments were cloned into a pGEM-T easy cloning vector (Promega, Madison, WI, USA). For Y2H constructs, BRI1 KS, fragments were digested with *Nde1*/*BamHI* restriction enzymes and cloned in the pGBKT7 vector (Clontech, Mountain View, CA, USA). For BiFC assays, full-length BRI1, BRI1LRR, and BRI1 kinase domains, as well as BAK1 full-length fragments were digested with *BamHI*/*Kpn1* restriction enzymes and cloned in pSPYNE or pSPYCE vectors. Specific primers used for cloning are listed in Table S1. All translational fusion constructs were verified by DNA sequencing. *HSP90.1* or *HSP90.2* were fused with C-terminal YFP [21], the full-length or truncated *BRI1 LRR* and *BRI1 KS*, and the full-length *BAK1* were fused with N-terminal YFP.

### 2.3. Y2H Assays

Interaction studies were performed in yeast strain SG335 using the Matchmaker GAL4 two-hybrid system according to the manufacturer’s protocol (Clontech, Mountain View, CA, USA). The full-length cDNAs of *HSP90.1* and *HSP90.2* were cloned into the pGADT7 vector (Clontech, Mountain View, CA, USA) as bait constructs [21] and full-length or truncated BAK1 or truncated forms of *BRI1* were cloned into the pGBKT7 vector (Clontech, Mountain View, CA, USA) as prey constructs using the appropriate primers (Table S1). Yeast transformants were tested on Synthetic Dropout (Clontech, Mountain View, CA, USA) medium -Leu, -Trp (SD-2), and interactions were assayed on selective -Leu, -Trp, and -His medium supplemented with 15 mM 3-amino-1, 2, 4-triazole (SD-3) (Sigma-Aldrich, St. Louis, MO, USA).

### 2.4. Protein Extraction and Western Blot Analysis

Proteins were extracted from hypocotyls of 5-day-old etiolated seedlings or 5-day-old etiolated seedlings of wild-type and mutant plants growing under control conditions or after treatments with chemicals using homogenization buffer 50 mM Tris-HCl pH 7.5, 5% glycerol, 150 mM NaCl, 0.1% NP-40, 1 mM PMSF (Sigma-Aldrich, St. Louis, MO, USA), 1xX PhosSTOP phosphatase inhibitor (Roche, Basel, Switzerland) and SIGMAFAST 1x protease inhibitor cocktail (Sigma-Aldrich, St. Louis, MO, USA). Equal amounts of total proteins were separated on an 8% SDS polyacrylamide gel either stained with coomassie brilliant blue (CBB) or transferred to Porablot PVDF membrane (Macherey-Nagel, Düren, Germany). Western blots were probed with anti-HSP90 sc-33755 (Santa Cruz Biotechnology, TX, USA), anti-BRI1 AS12 1859 (Agrisera, Vännäs, Sweden), and anti-BES1 [32]. The membrane was then washed and incubated with horseradish peroxidase-conjugated goat anti-rabbit IgG (Santa Cruz Biotechnology, Dallas, TX, USA). The chemiluminescence was detected using the Immobilon Crescendo Western HRP substrate (Millipore, MA, USA) followed by contemporary autoradiography techniques.

### 2.5. Immunoblot and Co-Immunoprecipitation Assays

Tobacco leaf cells transiently and singly expressing or co-expressing the HSP90.1–HA–YFPc or HSP90.2–HA–YFPc or BRI1LRR–cMyc–YFPn and BRI1 KS–cMyc–YFPn fusion proteins were harvested 3 days after infiltration. The tissue was ground in liquid nitrogen and total protein was extracted. The protein extracts were incubated overnight with anti-hemagglutinin (anti-HA) antibody and then with Protein G PLUS-Agarose (Santa Cruz Biotechnology, Dallas, TX, USA) at 4 °C for 4 h. The immunoprecipitated proteins and the corresponding protein extracts as inputs were resolved by SDS-polyacrylamide gel electrophoresis (SDS-PAGE) and detected by anti-c-Myc antibody and anti-HA antibody (Santa Cruz Biotechnology, Dallas, TX, USA).

### 2.6. BiFC and Epifluorescent Microscopy

BiFC assays were performed using *Agrobacterium*-mediated transformation. Fusion constructs in appropriate combinations were co-injected into *Nicotiana benthamiana* leaves. Tobacco mesophyll protoplasts were isolated and incubated in liquid medium as previously described [21]. At least three individual experiments were performed for each combination. BiFC protein–protein interactions were examined using epifluorescence microscopy 3 to 4 days after infiltration. YFP fluorescence was visualized using the fluorescent filter #41017, Endow GFP Bandpass Emission Filter (Chroma Technology Corp., Bellows Falls, VT, USA), and the chlorophyll autofluorescence was visualized with the U-MSWG filter set (Olympus BX-50 or Leica DMi8 inverted microscope with High-Speed Fluorescence—External Filter Wheels for Living Cell and Leica LAS X (version 3.7.2.22383) software). Images were taken with an Olympus DP71 camera, using Cell^A (Olympus Soft Imaging Solutions, Münster, Germany). Parameters for the signal detection were laser line 514 nm, emission spectrum 520–600 nm for GFP, and emission spectrum 610–660 nm for chlorophyll autofluorescence.

### 2.7. Confocal Microscopy

In vivo visualization of fluorescent reporters, such as *pHSP90.1:HSP90.1-GFP* and *pHSP90.2:HSP90.2-GFP* was performed with a confocal laser scanning microscope for membrane localization (Confocal Leica TCS SP5 on a DMI6000 Inverted Microscope). The cell patterns in roots of *pHSP90.1:HSP90.1-GFP* and *pHSP90.2:HSP90.2-GFP* expressing plants were delineated by the cell wall stain propidium iodide (PI; Thermo Fisher Scientific, Waltham, MA, USA). The images were captured under 488 nm laser excitation and 514 nm emission for the GFP channel and 586 nm laser excitation and 600 nm emission for the Propidium Iodide channel. Final merging of images was performed using Adobe Photoshop CS5 (version 9.01) software.

### 2.8. Quantification Relative Spatial Distribution at the PM in Nicotianna benthamiana Epidermal Cells

Live imaging was performed using a Leica DMi8 inverted microscope with High-Speed Fluorescence—External Filter Wheels for Living Cell and Software Leica LAS X (version 3.7.2.22383) Leica software (Leica, Wetzlar, Germany). *Nicotiana benthamiana* leaf samples were gently transferred between a glass slide and a cover slip in a drop of water. YFP and mCitrine (cYFP) fluorescence were observed with similar settings (i.e., excitation wavelength of 488 nm and emission wavelength of 490 to 550 nm). To obtain quantitative data, experiments were performed using strictly identical image acquisition parameters. Pseudo-colored images were obtained using the ‘Rainbow’ look-up-table (LUT) in Zen software. All quantifications were performed on raw images of at least 10 cells, at least two plants from each treatment, and at least three independent replicates.

For quantification of the relative spatial distribution at the PM, which reveals the degree of aggregation of fluorescence signal on the surface plane of the PM, fluorescence intensity was plotted with a 10 μm line on raw images of cells focused on the PM surface view. Three-line plots were recorded per cell at regions displaying high fluorescence intensity and three-line plots were recorded in regions displaying low fluorescence intensity. At least 15 cells per experiment were analyzed. For each plot, the relative spatial distribution at the PM was calculated as a ratio of the averages of high to low fluorescence intensities. All analyses were performed using ImageJ2.

### 2.9. Quantification of BRI1 and BAK1 Clustering Index

BRI1-GFP or BAK1-GFP signal was captured using Zeiss LSM880 with Airyscan mode under 488 nm laser excitation and 509 nm emission for the GFP channel and 586 nm laser excitation and 600 nm emission for the propidium iodide channel. The spatial clustering index was quantified as previously described with minor modifications [33]. In detail, in every individual experiment, all the samples were scanned using the same settings. The fluorescence signal profile at the PM was tracked by plotting the apical membrane to generate signal values along with a series of equidistant PM automatically. The fluorescence signal intensity of each plot was normalized to minimum and maximum values to create fluorescence signal values ranging within 0 to 1, to generate the clustering index. At least 10 cells were quantified, and more than 390 dots were automatically tracked in each cell. Thus, more than 4.000 points of relative signal intensity were obtained in each sample. Finally, the box plot diagrams of the GFP signal were created as averages of the fluorescence signal intensities from the equidistant series and show the spatial distribution of the BRI1 or BAK1 signal indicating the clustering index. The box plot diagrams were created in Microsoft Excel (2016).

### 2.10. Quantification and Statistical Analysis

In each case, at least three independent biological replicates were analyzed and similar results were obtained. Intra-assay variability was determined with technical triplicates. The details for all statistical parameters can be found in the figures or figure legends (type of statistical tests used; statistical significance denoted by asterisks). Error bars represent standard deviation as mentioned in the figure legends. Statistical analysis was performed using R (R version 4.0.3). A distribution analysis for each data set was done by a Lilliefors test and homogeneity was tested by a Levene’s test. For tests that involve pairwise comparisons including Western blot and RT-qPCR analyses, Student’s *t*-tests were used to determine if the differences were statistically significant. For multiple comparison tests, one-way ANOVAs were performed followed by Tukey’s tests to describe the levels of statistical significance. In cases where more parameters had to be taken into consideration, a two-way ANOVA followed by Tukey’s test was performed. Graphs were prepared using Microsoft Excel 2016. Statistical analysis results are presented in Supplementary Tables S2–S23.

### 2.11. Quantitative Analysis of Transcript Levels by RT-qPCR

Total RNA from 7-day-old etiolated seedlings of wild-type and mutant plants growing under control conditions or treated with BL was isolated following a phenol extraction protocol. RNA concentration and purity were determined before DNase I digestion with NanoDrop Lite (Thermo Scientific, Waltham, MA, USA). Reverse transcription and RT-qPCRs using specific primers were performed as previously described. *GAPDH* was used as a reference gene to calculate the relative gene expression. Experiments were performed in three biological replicates and the intra-assay variability was determined with technical triplicates. The primers are listed in Supplementary Table S1.

## 3. Results

### 3.1. BR Response in Etiolated Seedlings Is Channelled via HSP90

**Figure 1:**
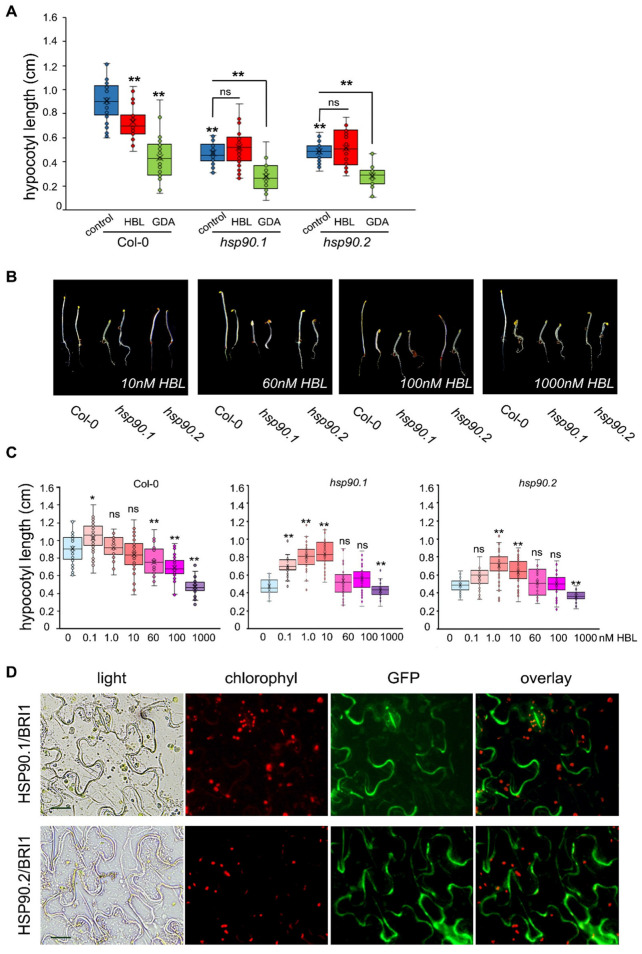
HSP90 is required for hypocotyl elongation. (**A**) Hypocotyl lengths sensitivity assays of 5-day-old etiolated Col-0, *hsp90.1,* and *hsp90.2*, seedlings grown in the presence of 60 nM HΒL or 2 μM GDA. (**B**) 5-day-old *hsp90.1* and *hsp90.2* seedlings grown in different concentrations of HBL. Left plant of each pair of seedlings shows growth under control conditions and right upon treatment with HBL. (**C**) Hypocotyl length sensitivity assays in 5-day-old etiolated Col-0, *hsp90.1,* and *hsp90.2* seedlings grown on a gradient of HBL concentrations. In box plots the middle line in the box represents median, the × shows mean, the bottom line depicts the 1st quartile, while the top line describes the 3rd quartile; the vertical lines (whiskers) extend to the minimum and maximum value within the 1.5× interquartile range (distance between the 1st and the 3rd quartile); points outside of the whiskers mark outliers (values outside of the 1.5× interquartile range). Data were analyzed by one-way ANOVA followed by Tukey’s test for multiple comparison (*): *p* < 0.05, (**): *p* < 0.01, ns: non-significant (n ≥ 30). In graph (**A**), the significance between the Col-0 at control conditions and the other treatment, Col-0 and *hsp90.1* or *hsp90.2* as well as between the control conditions of *hsp90* mutants and treatments are indicated. In graph (**C**), the significance between control conditions and treatments is presented for each genotype. (**D**) In vivo interactions between BRI1 and HSP90.1 or HSP90.2 were confirmed by BiFC assays in tobacco leaf epidermis Scale bars, 20 μm.

Mutations affecting BR hormone homeostasis and signaling influence hypocotyl elongation [3,34] through the BRI1-mediated signaling pathway [35]. To investigate the crosstalk of HSP90 and BR signaling in hypocotyl growth of etiolated seedlings, we studied the hypocotyl elongation of *hsp90.1* and *hsp90.2* mutants using homobrassinolide (HBL) or geldanamycin (GDA), a specific inhibitor of the activity of HSP90. In this study, we chose to work with T-DNA mutants of HSP90.1 and HSP90.2 cytosolic members, as the first one is a well-characterized heat shock inducible, while the second is the constitutively expressed member which has been described to have redundant and overlapping functions with the other two constitutively expressed HSP90.3 and HSP90.4 cytosolic members [25]. Under control conditions, both *hsp90.1* and *hsp90.2* etiolated seedlings showed significantly decreased hypocotyl elongation (Figure 1A and Figure S1A). Treatment with HBL resulted in a decrease of hypocotyl elongation of wild-type and *hsp90.2* mutant, while *hsp90.1* was insensitive to HBL (Figure 1A and Figure S1A). GDA decreased the hypocotyl elongation of *hsp90* mutants similarly to wild-type seedlings (Figure 1A and Figure S1A).

Concentrations of HBL higher than 10 nM, significantly decreased the hypocotyl growth of etiolated wild-type seedlings (Figure 1B,C and Figure S1B). Interestingly, under HBL concentrations up to 10 nM, the HBL dose response of hypocotyl growth was significantly increased in both *hsp90* mutants. However, a significant reduction of hypocotyl elongation was noticeable at the highest HBL concentration (Figure 1B,C and Figure S1B,C)

HSP90 acts as an activator for innate immune responses through toll-like receptors (TLRs), which have a protein architecture similar to BRI1 [36,37]. To address the involvement of HSP90 in the regulation of BL-mediated processes via the BRI1 receptor, we first investigated whether HSP90.1 and HSP90.2 are localized at the PM. Using HSP90.1 and HSP90.2 translational fusions [21], we confirmed their PM localization (Supplementary Figure S2A,B). Next, we tested the physical interactions between BRI1 and HSP90 in vivo. BiFC assays in *Nicotiana benthamiana* leaf epidermal cells and protoplasts, using the appropriate negative controls, verified the physical interactions between HSP90.1 and HSP90.2 with the BRI1 receptor (Figure 1D and Figure S2C,D).

### 3.2. LRR and the Kinase Domains of the BRI1 Receptor Interact Differently with HSP90.1 and HSP90.2

**Figure 2:**
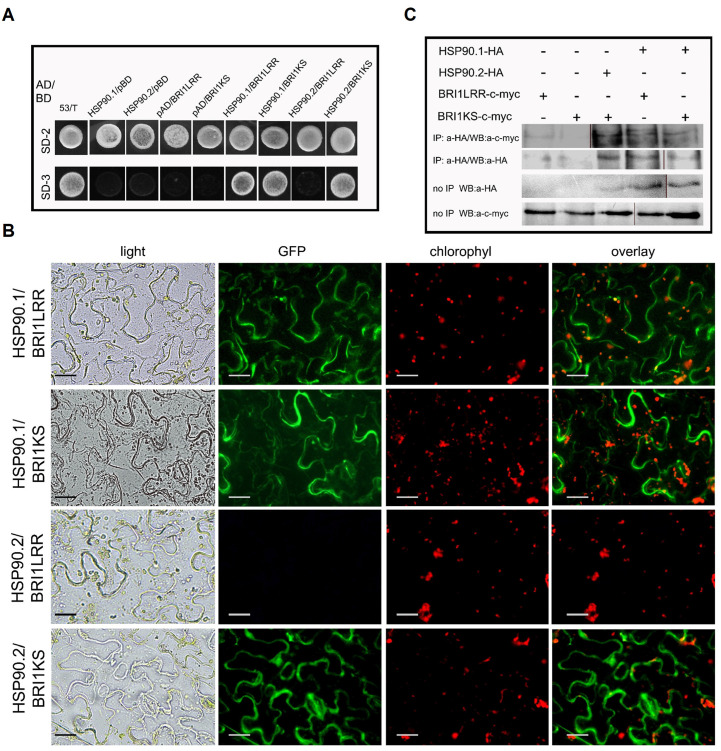
HSP90.1 and HSP90.2 interact with BRI1 receptor domains in vivo and in vitro. (**A**) Yeast two-hybrid assays using BRI1LRR and BRI1KS as baits and HSPSP90.1 or HSP90.2 as preys. Upper column, co-transformants grown on SD^-Leu-Trp^ medium (SD-2) demonstrate that both bait and prey plasmids are present in yeast cells. Positive selection of yeast two-hybrid interactions with minimal selection on SD^-Leu-Trp-His^ medium (SD-3). (**B**) Interactions between HSP90s and BRI1LRR or BRI1KS domains by BiFC assays in tobacco epidermis. Interaction was detected between HSP90.1 and BRI1LRR or BRI1KS, and between HSP90.2 and BRI1KS. Scale bars, 20 μm. (**C**) Co-immunoprecipitation assays of HSP90.1 with BRI1LRR and BRI1KS and HSP90.2 with BRI1KS. The co-immunoprecipitated proteins were detected by anti-c-Myc or anti-HA.

The extracellular domains of BRI1 (residues 1–784, BRI1LRR) and BAK1 (residues 1–220, BAK1LRR) are sufficient to form a BL-induced complex in insect cells [38]. To further explore the HSP90.1 and HSP90.2 interaction dynamics with the BRI1 receptor, we used the BRI1 domain (residues 1–689; BRI1LRR) encompassing the recognition brassinolide (BL) surface pocket embedded in a 70-aa island (residues 587–656) and the BRI1 kinase domain (residues 642–1192; BRI1KS) (Supplementary Figure S3A,B) [36,39,40] to perform yeast two-hybrid (Y2H) interaction assays, BiFC and co-immunoprecipitation experiments (Figure 2). BRI1LRR and BRI1KS constructs share a common region of 45 amino acid residues that correspond to the position of LRR20-24, the BR-ligand binding site (Supplementary Figure S3A).

The results of the Y2H assays showed that both HSP90.1 and HSP90.2 interact with BRI1KS, while only HSP90.1 interacts with BRI1LRR (Figure 2A). The BiFC assays of the HSP90s with the two domains of the BR receptor, conducted in *N. benthamiana* leaf epidermal cells and isolated protoplasts corroborated the results of Y2H revealing a signal at the periphery of the cell (Figure 2B and Figure S3D). The negative controls of BiFC assays showed no fluorescent signal (Supplementary Figure S3C,E). Co-immunoprecipitation (co-IP) assays using *N. benthamiana* leaves transiently expressing HSP90–HA–YFPc and BRI1LRR–cMyc–YFPn or BRI1KS-cMyc-YFPn verified the interactions with HSP90 (Figure 2C and Figure S10). The differences in the interactions of the two HSP90 members to the LRR and kinase domains of the BRI1 receptor combined with the visible hypocotyl elongation suggested a prominent role of the HSP90 molecular chaperones in the early events of BR signaling.

### 3.3. HSP90 Pharmacological Depletion Reduces the Levels of the BRI1 Receptor at the Plasma Membrane

**Figure 3:**
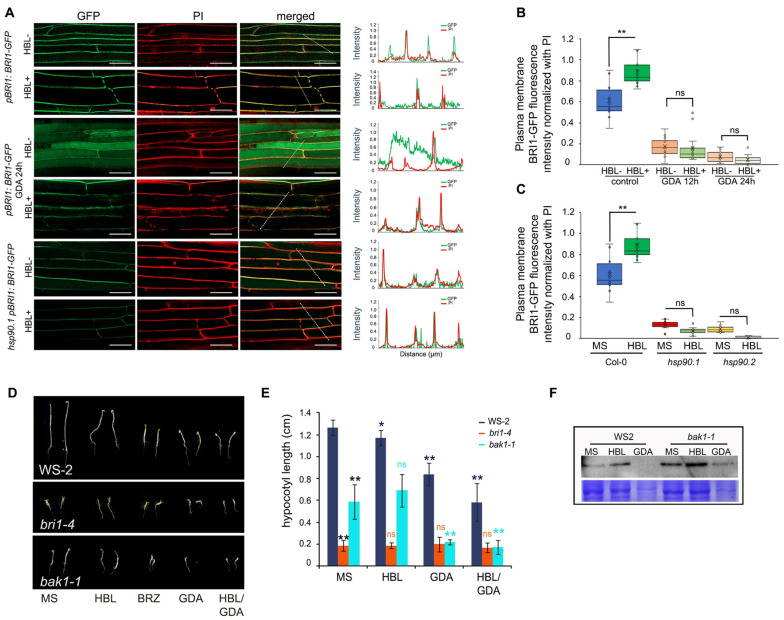
Impaired HSP90 function affects BRI1 protein levels at the plasma membrane of hypocotyl cells. (**A**) BRI1:BRI1-GFP localization in hypocotyl cells of 5-d-old, etiolated wild-type seedlings growing under control conditions or after treatment with 10 μΜ GDA for 24 h or *hsp90.1* mutant in resting conditions and upon the exogenous application of 100 nM HBL. The white lines mark the cells used for the quantification of the fluorescence signal intensity presented in plot profiles at the right of the images, generated by normalized values of fluorescence intensity. Propidium iodide (PI) cell wall stain was used to visualize the cell patterns in etiolated hypocotyls. Scale bars, 20 μm. (**B**,**C**) Quantification of relative plasma membrane (PM) fluorescence intensity of untreated and treated with 10 μm GDA BRI1-GFP hypocotyls (**B**) and *hsp90.1* and *hsp90.2* mutants (**C**)**,** in the absence or presence of 100 nΜ HBL. PΜ fluorescence intensity was normalized to background fluorescence for each measurement (n = 30). In box plots, the middle line in the box represents median, the × shows mean, the bottom line depicts the 1st quartile, while the top line describes the 3rd quartile; the vertical lines (whiskers) extend to the minimum and maximum value within the 1.5× interquartile range (distance between the 1st and the 3rd quartile); points outside of the whiskers mark outliers (values outside of the 1.5× interquartile range). Data were analyzed with ANOVA followed by Tukey’s test, ** is significant at *p* ˂ 0.01, ns is non-significant. (**D**) Images of 5-day-old etiolated WS-2, *bak1-1,* and *bri1-4* seedlings grown in the presence of 60 nM HΒL, 2 μM GDA and 60 nM HBL or 2 μM GDA. Scale bars, 0.5 cm. (**E**) Hypocotyl length sensitivity of 5-day-old etiolated WS-2, *bak1-1* and *bri1-4* seedlings grown under the indicated conditions. Data are presented as means ± SD (n > 30). Data were analyzed with one-way ANOVA followed by Tukey’s test, ** is significant at *p* ˂ 0.01, * is significant at *p* ˂ 0.05, ns is non-significant. (**F**) Western blot analysis using anti-BRI1 antibody. Proteins were extracted from 5-day-old etiolated seedlings of WS-2, *bri1-4,* or *bak1*-1 treated for 4 h with 60 nM HΒL or 2 μM GDA. CBB staining served as loading control.

Since HSP90 localizes at the PM and physically interacts with the BRI1 receptor, we tested the impact of HSP90 depletion on the subcellular localization of BRI1 under control conditions and during HBL activation. Under control conditions, the BRI1 receptor localized, as expected, at the PM of hypocotyl cells in 5-day-old etiolated seedlings (Figure 3A). Both pharmacological and genetic inhibition of HSP90 activity decreased the levels of BRI1-GFP at the PM and induced its accumulation in the intracellular space (Figure 3A and Figure S4A,B). Quantification of the fluorescence signal intensity of PM localized BRI1 receptor in the hypocotyl cells of etiolated seedlings under control conditions or after treatment with HBL, or upon GDA application and treatment with HBL supported the observations made at the microscope (Figure 3B). Notably, the quantification of fluorescence signal intensity in HBL-treated wild-type seedlings showed that BRI1-GFP fluorescence intensity increased significantly at the PM, while pharmacological or genetic depletion of HSP90 showed no significant response to HBL suggesting that HSP90 activity is required for normal response to HBL (Figure 3A–C and Figure S4A,B).

BAK1 forms heterodimers with BRI1 to transduce the BR signal in a phosphorylation-mediated cascade of transcriptional programs [3,40]. To determine whether HSP90 inhibition similarly affects the BAK1 co-receptor we measured the hypocotyl length of 5-day-old dark-grown seedlings in control medium and in medium containing 60 nM HBL, 2 μΜ GDA, and both 60 nM HBL and 2 μΜ GDA of wild-type (WS2), as well as of *bri1-4* and *bak1-1* mutants. Interestingly, *bri1-4* seedlings were insensitive to all the treatments. In contrast, *bak1-1* seedlings were sensitive to GDA and HBL/GDA treatments (Figure 3D,E). Western blot analysis of 5-day-old etiolated seedlings grown οn plain MS or MS supplemented with 60 nΜ HBL or 2 μΜ GDA showed that HSP90 depletion results in reduced BRI1 protein levels, corroborating the observations made at the microscope (Figure 3F and Figure S10). Altogether, our results strongly support the notion that HSP90 activity regulates the subcellular localization, the recycling, and the abundance of the BRI1 receptor at the PM.

### 3.4. Genetic Depletion of HSP90 Leads to Impaired BR Signaling Downstream of BRI1 and BAK1 Receptors

Analysis of the phosphorylation levels of the BES1 transcription factor showed that HBL treatment of wild-type plants decreased the phosphorylation levels of BES1. GDA treatment increased the phosphorylated pools of the BES1 transcription factor supporting previous findings [24]. Increased phosphorylation of BES1 was found in both *bri1-4* and *bak1-1* mutants (Supplementary Figures S5A,B and S10).

To determine the role of HSP90.1 and HSP90.2 downstream of the BRI1 and BAK1 receptors, we also investigated the phosphorylation of the BES1 transcription factor in 5-day-old etiolated seedlings of wild-type, *hsp90.1* and *hsp90.2* grown under control conditions or in the presence of 60 nM HBL or 2 μM GDA. In the *hsp90.2* mutant, the phosphorylation of BES1 was significantly decreased under control conditions. No response was observed in treatments with HBL or GDA in either *hsp90.1* and *hsp90.2* mutants (Supplementary Figures S5C,D and S10).

Analysis of the transcript levels of well-characterized transcriptional targets of the BR signaling cascade [41] such as *DWARF4* (*DWF4*), *CONSTITUTIVE PHOTOMORHOGENESIS AND DWARFISM* (*CPD*), and *TOUCH4* (*TCH4*) in 5-day-old wild-type etiolated seedlings, *hsp90.1* and *hsp90.2* mutants showed downregulation of the biosynthetic genes *DWF4* and *CPD* and upregulation of *TCH4* in HBL-treated wild-type plants (Supplementary Figure S5E–G). HBL application increased the transcription of *DWF4* and *CPD* genes in *hsp90.1*, while it had no impact on the transcription of *DWF4* and *CPD* in *hsp90.2*, compared with the wild-type seedlings (Supplementary Figure S5E). The expression levels of *CPD* and *TCH4* were downregulated in both *hsp90.1* and *hsp90.2* mutants in control conditions (Supplementary Figure S5F,G). Nevertheless, *TCH4* was upregulated in BL-treated *hsp90.1* seedlings and downregulated in *hsp90.2* HBL-treated seedlings (Supplementary Figure S5G). These data suggest that HSP90 inhibition leads to the attenuation of BR signaling through the phosphorylation and inactivation of the BES1 transcription factor altering the expression of its transcriptional targets.

### 3.5. HSP90 Interacts with BAK1 Co-Receptor and Regulates Its Levels at the Plasma Membrane

**Figure 4:**
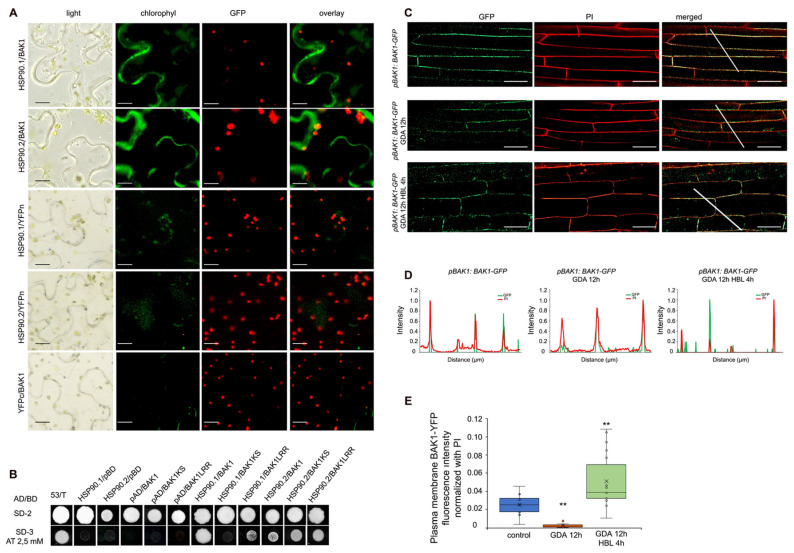
HSP90 physically interacts with the BAK1 co-receptor and modulates its abundance at the plasma membrane. (**A**) BiFC interaction assays of HSP90.1 or HSP90.2 with BAK1 in *N. benthamiana* leaf epidermal cells. Scale bars, 20μm. (**B**) Yeast two-hybrid assays using full-length BAK1, BAK1LRR, or BAK1KS as baits and HSPSP90.1 or HSP90.2 as preys. Upper column, co-transformants grown on SD^-Leu-Trp^ medium (SD-2) demonstrate that both bait and prey plasmids are present in yeast cells. Positive selection of yeast two-hybrid interactions with minimal selection on SD^-Leu-Trp-His^ medium (SD-3) supplemented with 2,5 mM 3-amino-1,2,4-triazole (3-AT). (**C**) BAK1:BAK1-GFP localization in hypocotyl cells of 5-d-old wild-type etiolated seedlings growing under normal conditions or after treatment with 10 μΜ GDA for 12 h and 24 h in resting conditions and upon the exogenous application of 100 nM HBL. The white lines mark the cells used for the quantification of the fluorescence signal intensity. Propidium iodide (PI) cell wall stain was used to visualize the cell patterns in etiolated hypocotyls. Scale bars, 20 μm. (**D**) Plot profiles generated by normalized values of fluorescence intensity. (**E**) Quantification of fluorescence signal intensity of BAK1-GFP in the hypocotyl cells of GDA-treated and untreated 5-day-old etiolated seedlings (n > 30). Box plots show the first and third quartiles, split by the median (line) and mean (cross). Data are analyzed by one-way ANOVA followed by Tukey’s test; ** *p* < 0.01. The asterisks indicate the significance between the treatments and the control.

Next, we sought to investigate whether HSP90 also interacts with BAK1. BiFC interaction assays in *N. benthamiana* epidermal cells, transiently expressing HSP90.1 or HSP90.2 with BAK1 (Figure 4A) showed that both HSP90.1 and HSP90.2 interact with the BAK1 co-receptor, while there was no fluorescent signal in the appropriate negative controls (Figure 4A). Analysis of HSP90 and BAK1 interaction dynamics showed that HSP90.1 interacts only with the LRR (1–689 amino acid residues) domain of the BAK1 receptor. In contrast, HSP90.2 interacts with both the LRR and kinase (642–1192 amino acid residues) domains of BAK1 in Y2H assays (Figure 4B).

The impact of HSP90 pharmacological inhibition on the PM localization of BAK1 in 5-day-old plants expressing the *pBAK1*:BAK1-GFP transgene, showed that GDA treatment significantly decreased the abundance of BAK1-GFP at the PM compared to the resting conditions. In contrast to the BRI1 PM localization, the levels of BAK1-GFP at the PM were increased in hypocotyl cells of etiolated seedlings treated with BL upon HSP90 inhibition with GDA (Figure 4C–E) suggesting that HSP90 function regulates the levels of BAK1 at the PM during BR signaling.

### 3.6. Impaired HSP90 Function Alters the Spatial Dynamics of the BRI1 Receptor at the Plasma Membrane

The BRI1 receptor is heterogeneously distributed at the PM, organized in functional microdomains, while BR regulates the activity and dynamic behavior of BRI1 particles [42]. Alterations in the lateral spatial organization of proteins at the PM have been linked with increased protein endocytosis [43,44].

**Figure 5:**
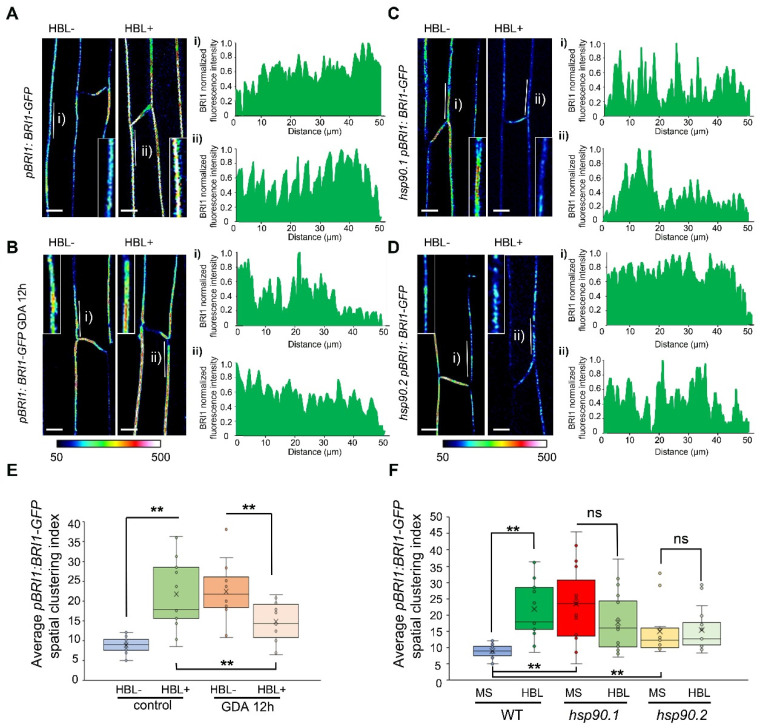
HSP90 function controls the spatial dynamics of BRI1 receptor at the plasma membrane modulating BR response. (**A**–**D**) LUT images representing a quantitative fluorescence intensity evaluation of BRI1-GFP expression levels in the indicated treatments and genotypes using a pseudo-color-coded range from the scale, where dark blue represents zero intensity (50 in arbitrary units) and white represents maximum intensity (500 in arbitrary units). The white lines mark the PM region used for the quantification of the fluorescence signal intensity to generate the BRI1 clustering index presented in plot profiles at the right of the images. Insets show magnifications of the region of PM used for quantification. Plot profiles (**i**) and (**ii**) correspond to cells residing in the areas marked with white lines. Propidium iodide (PI) cell wall stain was used to visualize the cell patterns in etiolated hypocotyls. Scale bar: 10 μm. (**E**) Quantification of the average spatial clustering index of BRI1 at the PM of etiolated hypocotyl cells of treated and untreated seedlings with 100 nM HBL for 4 h or after pretreatment with 10 μΜ GDA for 12 h. (**F**) Quantification of the average spatial clustering index of BRI1 at the PM of etiolated hypocotyl cells of treated and untreated seedlings with 100 nM HBL for 4 h of the indicated genotypes. In box plots, the middle line in the box represents median, the × shows mean, the bottom line depicts the 1st quartile, while the top line describes the 3rd quartile; the vertical lines (whiskers) extend to the minimum and maximum value within the 1.5× interquartile range (distance between the 1st and the 3rd quartile); points outside of the whiskers mark outliers (values outside of the 1.5× interquartile range). The data were analyzed with one-way ANOVA followed by Tukey’s test, statistically significant differences compared to control are shown, ** is significant at *p* ˂ 0.01, ns—not significant. Asterisks indicate the significance between the pair of treatments compared.

Our results demonstrated a critical and distinctive role of HSP90 in the regulation of BRI1/BAK1 abundance at the PM, pointing to an intertwined activation of the receptors in the presence of the appropriate stimulus. In this context, we analyzed the profiles of BRI1-GFP signal intensity at the PM of 5-day-old etiolated seedlings treated with HBL after the application of GDA. In agreement with previous findings [42], BRI1-GFP fluorescence showed distinct foci of signal distribution at the PM under resting conditions, as evidenced by the qualitative presentation of the fluorescence intensity (Figure 5A and Figure S6A). Application of HBL increased the intensity and altered the distribution of BRI-GFP at the PM (Figure 5A,E and Figure S6B,I), while application of GDA significantly decreased the BRI1-GFP fluorescent intensity at the PM and altered its distribution (Figure 5B,E and Figure S6C,I). To investigate the impact of the depleted function of HSP90 in BR response, we applied HBL in etiolated wild-type seedlings pretreated with GDA. Our findings showed that HBL application upon GDA treatment significantly induced the heterogeneous distribution of BRI1-GFP foci at the PM (Figure 5B and Figure S6D).

Genetic depletion of HSP90.1 or HSP90.2 revealed decreased signal intensities of BRI1-GFP at the PM of hypocotyl cells of untreated and treated with HBL *hsp90.1* and *hsp90.2* etiolated seedlings (Supplementary Figure S6E–H,J). Significant changes in BRI1-GFP distribution were also observed (Figure 5C,D).

The spatial clustering index (SCI) in equidistant fluorescence intensity profiles [33] was used to reveal the segregation of fluorescent signal and to describe the role of HSP90 in the lateral organization of BRI1 at the PM. Analysis of the SCI supported the results described above (Figure 3, Figure 5 and Figure S6I,J), because HBL treatment increased the SCI of BRI1-GFP, suggesting increased clustering. Application of HBL in etiolated seedlings pre-treated with GDA caused a small though significant decrease in the SCI (Figure 5E) suggesting perturbations in BR response. Based on the SCI analysis, we observed an induction of BRI1-GFP clusters at the PM of both *hsp90.1* and *hsp90.2* etiolated hypocotyl cells (Figure 5F), even though the intensity was decreased. No effect was detected for HBL-treated etiolated seedlings (Figure 5F and Figure S6J). Therefore, our data suggest that HSP90 is genetically required for the control of the distribution of the BRI1 receptor at the PM of etiolated seedlings.

Next, we tested the effect of HSP90 inhibition on the spatial distribution of BAK1 at the PM. We showed that BAK1-GFP is also heterogeneously organized at the PM in resting conditions (Supplementary Figure S7A,B). Treatment with GDA significantly decreased the BAK1-GFP fluorescent intensity and altered its SCI at the PM (Supplementary Figure S7). Even though the fluorescence signal intensity of both BRI1 and BAK1 significantly decreased upon HSP90 inhibition, the lateral organization of ΒAΚ1 changed in an inverse direction exhibiting a more dispersed and labile structure (Supplementary Figure S7E,F). HSP90 is crucial in BRI1/BAK1 abundance and distribution at the PM which is an important parameter mediating BR signaling.

### 3.7. HSP90.1 Mediates BRI1/BAK1 Interactions

**Figure 6:**
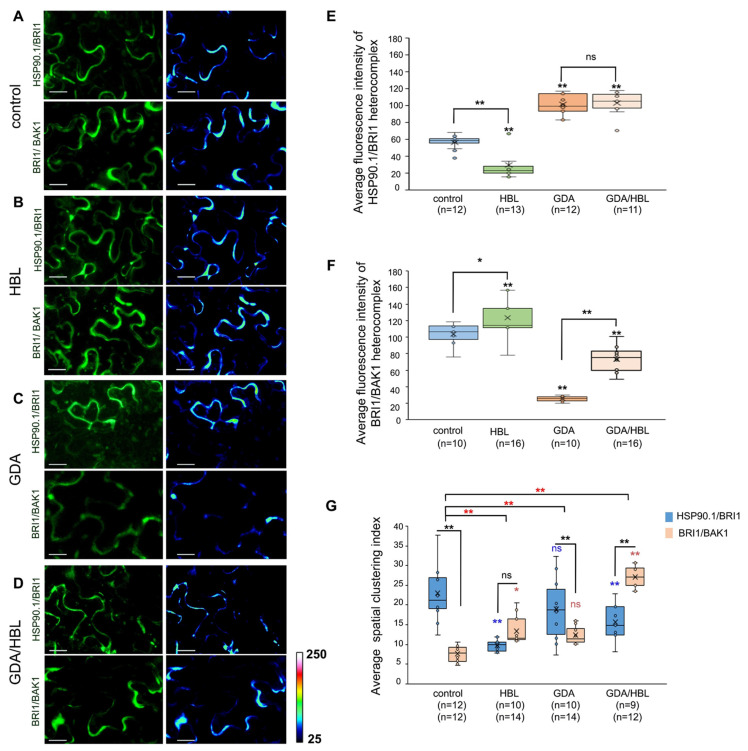
The association of HSP90.1 with the BRI1 receptor affects the activation of BRI1/BAK1 heterocomplex upon HBL perception. (**A**–**D**) BiFC interaction assays in tobacco leaf epidermis of HSP90.1 with BRI1 and BRI1 with BAK1 under control conditions and after treatments with 1 μM HBL for 4 h or upon treatment with 10 μM GDA for 12 h followed by application of 1 μΜ of HBL for 4 h. From left to right: fluorescent YFP signals, and semi-quantitative fluorescence intensity evaluation of HSP90.1/BRI1 or BRI1/BAK1 levels using pseudo-color-coded range from the scale, where dark blue represents zero intensity (25 in arbitrary units) and white represents maximum intensity (250 in arbitrary units). (**E**) Quantification of the average fluorescence intensity of HSP90.1/BRI1, heterocomplex at the PM under the indicated conditions. Three different areas of low and high fluorescence intensity were measured per cell. (**F**) Quantification of the average fluorescence intensity of BRI1/BAK1 heterocomplex at the PM under the indicated conditions. (**G**) Quantification of the relative spatial distribution of HSP90.1/BRI1and BRI1/BABK1 complexes at the PM under the indicated conditions. n, the number of cells analyzed in each treatment. Box plots show the first and third quartiles, split by the median (line) and mean (cross). Data are analyzed by one-way ANOVA followed by Holm’s test; * *p* < 0.05, ** *p* < 0.01, ns—non-significant. The red stars in the diagram show the statistically significant differences between the treatments analyzed by two-way ANOVA. Scale bars, 25 μm.

The application of BL promotes the binding of BRI1 to its co-receptor BAK1 resulting in the propagation of the downstream signaling [45,46]. To elucidate the role of HSP90 in the association of BRI1 with BAK1 we investigated if the presence of HBL affects the interaction of HSP90.1 with the BRI1 receptor. Quantification of the fluorescence intensity in BiFC assays under control conditions and HBL treatment showed significant a reduction in the reconstructed GFP signal, suggesting that HBL treatment weakened the binding of BRI1 to HSP90.1 (Figure 6A,B,E and Figure S8B). Next, we investigated whether HSP90 inhibition with GDA is involved in the interaction of HSP90.1 with BRI1. Interestingly, we observed that GDA treatment did not affect HSP90.1/BRI1 complex formation and it rather stabilized HSP90.1/BRI1 heterocomplex (Figure 6A,C,E and Figure S8C). HBL application in GDA-treated tobacco leaves did not cause any significant changes in the fluorescence intensity of the heterocomplex (Figure 6D,E and Figure S8D).

We assessed the interaction intensity of BRI1/BAK1 and we showed that HBL treatment increased the interaction between BRI1 and BAK1 in agreement with previous findings [42]. Application of GDA resulted in a weak interaction between them (Figure 6A–C,F and Figure S8A–C). Treatment with HBL in the presence of GDA reinforces the formation of BRI1/BAK1 heterodimer (Figure 6A,D,F and Figure S8A,D). Consistent with these results, pharmacological inhibition of HSP90 also abolishes the association of HSP90.2 with the BRI1 receptor, even though under control conditions the intensity of the HSP90.2/BRI1 interaction was significantly greater than that of HSP90.1/BRI1 and comparable to BRI1/BAK1 (Supplementary Figures S8E and S9). Our results suggest that BRI1/BAK1 heterodimerization depends on the activity of HSP90. 

We next asked how HBL application under resting conditions or upon treatment with GDA impacts the lateral organization of HSP90.1/BRI1 and BRI1/BAK1 at the PM. Quantification of the SCI showed that there was a significantly different spatial organization between HSP90.1/BRI1 and BRI1/BAK1 at the PM under control conditions, as the HSP90.1/BRI1 complex is organized in distinct, clustered structures compared to that BRI1/BAK1 complex that exhibits a more disperse organization at the PM (Figure 6G). However, we observed that the spatial dynamics of HSP90.2/BRI1 was similar to BRI1/BAK1 and different from the HSP90.1/BRI1 heterocomplex (Supplementary Figures S8F and S9). Interestingly, HBL treatment resulted in profound alterations in the spatial organization of both HSP90.1/BRI1 and BRI1/BAK1 heterocomplexes (Figure 6G). Despite the different localization trends, it is clear that the two heterocomplexes had similar distribution patterns at the PM. Notably, GDA treatment did not significantly change the spatial dynamics of HSP90.1/BRI1 or BRI1/BAK1 at the PM and retained the significantly different spatial organization between the two heterocomplexes which is similar to that observed under the control conditions. Nevertheless, HBL application in GDA-treated tobacco leaves resulted in significant alterations of the spatial organization at the PM of both HSP90.1/BRI1 and BRI1/BAK1 heterocomplexes, suggesting spatial sequestration that could indicate functional separation (Figure 6G). Our findings suggest that the association of HSP90 with BRI1 modulates the partitioning of the BRI1 receptor at the PM, and the chaperone activity is decisive for the partitioning of the BRI1/BAK1 heterodimer formation in the presence of active BR signaling.

## 4. Discussion

In addition to their role in the maturation of kinases [16,17,29,47], HSP90 acts as a molecular scaffold regulating the subcellular localization and translocation of signaling proteins within the cell providing an organization network to regulate signal transduction and directional growth [45,46,47,48,49,50,51,52]. However, the role of plant HSP90 molecular chaperone in signaling processes is emerging, highlighting differences and similarities between the plant and animal kingdoms [18]. The action of HSP90 in coupling signaling cascade partners to modulate the activation of signaling pathways was revealed by its role in the regulation of stomatal proliferation and differentiation, embryo development, and circadian clock-controlled developmental processes [53,54,55,56].

HSP90 binding to diverse proteins including PM proteins has been linked to different cellular outputs depending on the type of the client protein that it associates with [17,27,57,58,59,60]. Our data showed that the reduction in active HSP90 levels controls the abundance and localization pattern of BR receptors at the PM. Genetic and pharmacological depletion of HSP90 revealed accumulation of BRI1 within the intracellular space indicating that the endocytosis mechanisms that maintain the dynamic recycling of BRI1 are affected by the HSP90. Analysis of the interaction between BRI1/BAK1 revealed changes in the spatial distribution of the receptor/co-receptor complex upon GDA application in the presence of the BL ligand. The HSP90 homodimer configuration depends on ATP binding and this conformation could have distinct clients [61].

The subcellular localization of a protein is tightly connected with its function and determines the accessibility to interact with specific partners or its integration into functional signaling cascades. It is suggested that the spatial heterogeneity of protein complexes provides a mechanistic process by which signaling cascades can be sequestered in distinct cellular compartments [62]. Partitioning at the PM of membrane-bound proteins is considered to improve signaling reliability as it moderates the nonlinearity of the switching process and reduces the noise of the response [63]. The efficiency of signaling transduction depends on the selective accumulation of signaling proteins into discrete clusters at the PM. Recruitment of signaling proteins into specialized microdomains [64,65] could mediate the spatiotemporal concentration of specific signaling molecules to properly activate developmental processes [66,67]. In Arabidopsis, HSP90 was found to interact with flotillin 2, a PM protein found in membrane microdomains [68] and BRI1 resides in nanoclusters that are marked by FLOT1 [42]. In animal systems, HSP90 alters the composition of signaling partners in PM microdomains, affecting the downstream signaling process [50].

BR promotes the partitioning of BRI1 into functional membrane microdomains to activate BR signaling [42]. The role of HSP90 in the sequestration of signaling components in PM microdomains, such as lipid crafts, has been reported in the desensitization of receptors and during heat stress response in animal systems [69,70]. Genetic or pharmacological depletion of HSP90 decreased the abundance of BRI1 and BAK1 proteins at the PM in 5-day-old etiolated seedlings and induced the internalization of the receptors. Moreover, inhibition of HSP90 weakened the BRI1 interaction with BAK1 and altered the spatial distribution of both BRI1 and BAK1 receptors at the PM in a different manner, as it induced BRI1 accumulation in clusters and resulted in a more dispersed organization in the case of BAK1. Under resting conditions, a comparison of the spatial distribution showed differences between the localization of the HSP90.1/BRI1 complex and BRI1 associated with BAK1. However, a similar organization was detected between the HSP90.2/BRI1 and BRI1/BAK1 heterocomplexes. These data indicate that HSP90 adjusts BRI1 and BAK1 membrane distribution and abundance, and the dynamic distribution of BRI1/BAK1 at the PM is HSP90 dependent. During BR activation, the interaction of HSP90.1 with BRI1 was weaker compared with the association of BRI1 with BAK1. However, the two heterocomplexes exhibited similar lateral organization at the PM, suggesting that HSP90 facilitates the formation of active ligand-perceiving receptor complexes between BRI1 and BAK1.

The role of HSP90 in the association of BRI1 with BAK1 at the PM is further supported by previous studies that analyzed the mode of function of TWD1, a co-chaperone of HSP90, in the association of BRI1 and BAK1 [71,72]. The activity of TWD1 is critical for the autophosphorylation of the two receptors after the ligand binding and the maintenance of the BAK1 co-receptor in a signaling-competent state at the PM. The presence of the hyperphosphorylated forms of BZR1 in *twd1* mutants indicates a positive role for TWD1 in BR signaling upstream of BIN2 [71]. Thereby, TWD1 activity mediates the linking of different signaling pathways facilitating protein–protein interactions within functional networks [73]. Our findings also suggest a similar role for HSP90 in the formation of BRI1 and BAK1 complexes.

**Figure 7:**
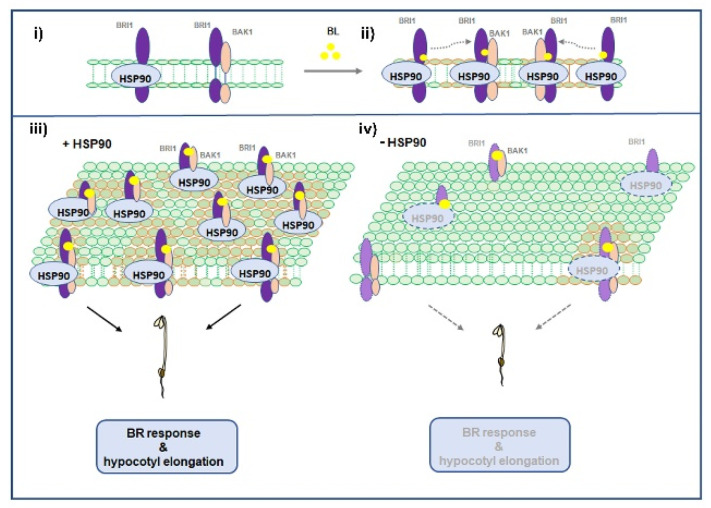
Proposed model for the function of HSP90 in the activation of BRI1 at the plasma membrane. In resting conditions, BRI1 associates with HSP90.1, and this interaction prevents the association of BRI1 to BAK1, as it was demonstrated by the sequestration of the two complexes at the PM. HSP90.1 association with BRI1 keeps the latter poised for activation, since in the presence of ligand (BL) HSP90.1-bound BRI1 incorporates in the BRI1/BAK1 heterocomplexes, which is deduced by their similar PM localizations. Interaction assays of HSP90.1 and BAK1 with truncated forms of the BRI1 receptor showed that although BRI1 kinase activity does not seem to depend on HSP90 ATPase function, it modulates the changes in the partitioning of HSP90.1/BRI1 and BRI1/BAK1 complexes at the PM and mediates the integration of HSP90.1 bound BRI1 into a complex with BAK1 co-receptor. (**i**) In the absence of the active ligand HSP90 interacts with BRI1, while partitioning of HSP90-bound BRI1 receptor is distinct from the partitioning of BRI receptor associating with its co-receptor BAK1 at the plasma membrane. (**ii**) Upon ligand activation the plasma membrane distribution of both HSP90-bound BRI1 and BAK1 associating BRI1 are similar suggesting the incorporation of HSP90-bound BRI1 to activated heterocomplexes with BAK1. (**iii**) In the presence of HSP90, ligand activation leads to increased abundance of BRI1 and to symmetric distribution of the activated BRI1-BAK1 heterocomplexes at the plasma membrane, which mediates the initiation of the downstream signaling cascade that controls BR response. (**iv**) Upon depletion of HSP90, the presence of active ligand does not increase the levels of BRI1 at the plasma membrane and the spatial sequestration of HSP90-bound BRI1 from the BRI1/BAK1 heterocomplexes at the plasma membrane, is enhanced leading to the attenuation of the downstream BR signaling and response.

Overall, our results suggest a dynamic bipartite role of HSP90 in the activation of BRI1 upon ligand binding and the subsequent association with its co-receptor, BAK1, as summarized in the proposed model (Figure 7). Our analysis underlines the HSP90-mediated regulation of the spatial distribution and accumulation of BRI1 and BAK1 receptors at the PM. The depletion of HSP90 impacts the BRI1 or BAK1 receptor pool at the PM and triggers a heterogeneous distribution of membrane-bound BRI1, thus preventing their association, which attenuates BR signaling and results in the reduction in hypocotyl elongation in etiolated seedlings. Cell elongation, a BRI1-mediated developmental process, was found to depend on HSP90 function, as hypocotyl elongation of *hsp90* etiolated seedlings resembled that of BL-treated or BRI1 overexpressing plants [35]. Our findings demonstrate a critical activity of HSP90 in the formation of a scaffold that acts as a platform that mediates interactions between the activated receptors BRI1 and BAK1 resulting in advancing downstream signaling components to efficiently regulate signal transduction. Multiple reports have highlighted the important role of the brassinosteroid signaling pathway to stress tolerance and adaptation to heat stress [74,75]. Understanding the precise role of the HSP90 molecular chaperone in fine-tuning the BR hormonal pathway could open new perspectives to improve plants’ tolerance and acclimation to stress conditions.

## Data Availability

All relevant data can be found and are available within the manuscript and supporting information.

## Supplementary Materials

The following supporting information can be downloaded at: https://www.mdpi.com/article/10.3390/cells11213341/s1; Figure S1: HSP90 is involved in BR response; Figure S2: HSP90.1 and HSP90.2 reside at the PM; Figure S3: BRI1LRR and BRI1 KS interact differently with HSP90.1 and HSP90.2; Figure S4: BRI1 localization in hypocotyl cells of *hsp90.2*; Figure S5: HSP90 regulates the transcriptional activation of BES1; Figure S6: Images used for the analysis of BRI1 spatial clustering index of wild-type and hsp90 mutants after treatment with BL and under control conditions; Figure S7: HSP90 regulates the spatial distribution of BAK1 co-receptor at the PM; Figure S8: Images of BiFC interaction assays of HSP90.1 with full-length BRI1 protein or BRI1 with BAK1; Figure S9: HSP90.2 does not interact with BRI1 receptor in the presence of GDA; Figure S10: Unprocessed Western blot images used in this study; Table S1: Primers list; Table S2: Statistical analysis of Figure 1A; Table S3: Statistical analysis of Figure 1C (Col-0); Table S4: Statistical analysis of Figure 1C (*hsp90.1*); Table S5: Statistical analysis of Figure 1C (*hsp90.2*); Table S6: Statistical analysis of Figure 3B; Table S7: Statistical analysis of Figure 3C; Table S8: Statistical analysis of Figure 3E; Table S9: Statistical analysis of Figure 4E; Table S10: Statistical analysis of Figure 5E; Table S11: Statistical analysis of Figure 5F; Table S12: Statistical analysis of Figure 6E; Table S13: Statistical analysis of Figure 6F; Table S14: Statistical analysis of Figure 6G; Table S15. Statistical analysis of Figure S1C (Col-0); Table S16. Statistical analysis of Figure S1C (*hsp90.1*); Table S17. Statistical analysis of Figure S1C (*hsp90.2*); TableS18: Statistical analysis of Figure S5B; TableS19: Statistical analysis of Figure S5D; Table S20. Statistical analysis of Figure S5E; Table S21. Statistical analysis of Figure S5F; Table S22. Statistical analysis of Figure S5G; Table S23. Statistical analysis of Figure S6I; Table S24. Statistical analysis of Figure S6J; Table S25. Statistical analysis of Figure S7E; Table S26. Statistical analysis of Figure S7F; Table S27. Statistical analysis of Figure S8E; Table S28. Statistical analysis of Figure S8F.
