# Supplementary material for: BRI1 and BAK1 Canonical Distribution in Plasma Membrane Is HSP90 Dependent
Source: Cells. 2022 Oct 22;11(21):3341. doi: 10.3390/cells11213341 (PMC9656807; doi:10.3390/cells11213341)
Supplement: Supplementary file 1 [file cells-11-03341-s001.zip › cells-1964341-supplementary.pdf]

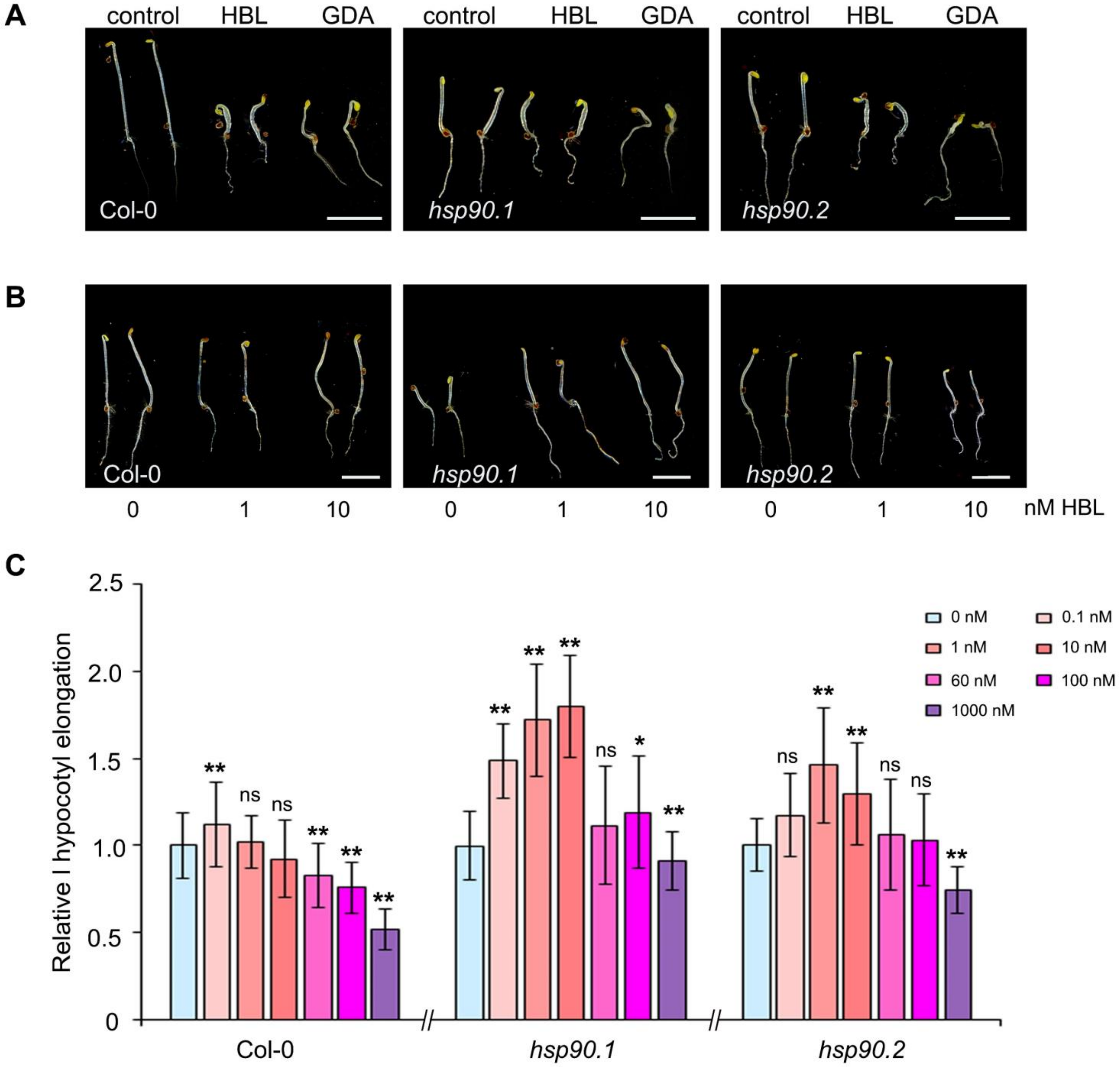

Supplementary Figure S1. HSP90 is involved in BR response. (**A**) Images of 5-day-old etiolated *Col-0*, *hsp90.1* and *hsp90.2* seedlings grown on 60nm HBL or 2μm GDA. (**B**) Images of 5-day-old etiolated Col-0, *hsp90.1* and *hsp90.2* seedlings grown on MS, and MS supplemented with 0.1 or 10 nM HBL. Scale bars: 0.5 cm. (C) Relative hypocotyl elongation. The values of hypocotyl lengths of Col, *hsp90.1* and *hsp90.2* seedlings after treatment with various concentrations of HBL, were normalized to the hypocotyl lengths of the corresponding 5-day-old seedlings growing at control conditions. In box plots, the middle line in the box represents median, the × shows mean, the bottom line depicts the 1st quartile, while the top line describes the 3rd quartile; the vertical lines (whiskers) extend to the minimum and maximum value within the 1.5× interquartile range (distance between the 1st and the 3rd quartile); points outside of the whiskers mark outliers (values outside of the 1.5× interquartile range). The data were analysed with one-way ANOVA followed by Tukey's test, statistically significant differences compared to control are shown, ** significant at p<0.01, * significant at p<0.05, ns- not significant.

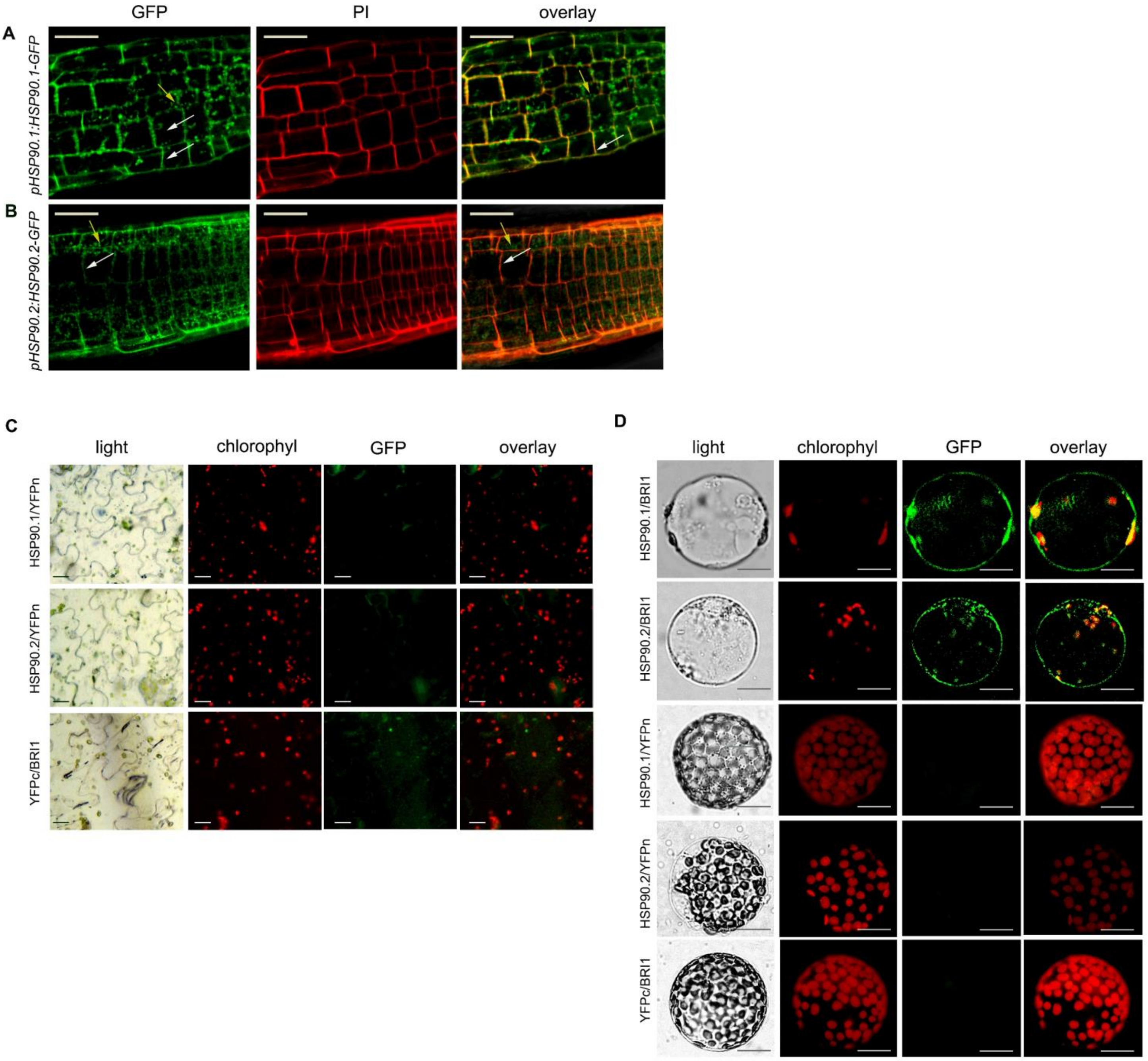

Supplementary Figure S2. HSP90.1 and HSP90.2 reside at the PM. (**A-B**) Confocal microscope images of root epidermal cells of transgenic Arabidopsis seedlings expressing *HSP90.1-GFP* and *HSP90.2-GFP*. HSP90.1-GFP (a) and HSP90.2-GFP (b) (green signal) localize at the periphery of the cell (white arrows), PI (red signal) was used for the staining of cell walls. (**C**) Negative controls of BiFC interactions assays of HSP90.1 or HSP90.2 with BRI1. HSP90.1 pSPYCE (HSP90.1) or HSP90.2 pSPYCE (HSP90.2) co-expressed with pSPYNE vector, or pSPYCE vector co-expressed with BRI1 pSPYNE (BRI1) in tobacco epidermis cells. **(D)** *In vivo* interactions between BRI1 and HSP90.1 or HSP90.2 were confirmed by BiFC assays in tobacco protoplasts. Scale bars: 20 μm.

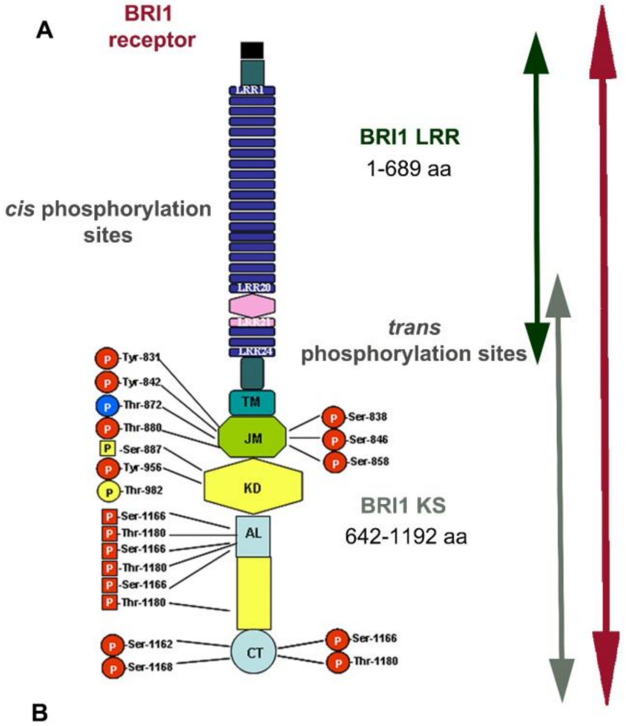

B

CELLO Prediction Results	BRI1 LRR	BRI1 KS
Extracellular	2.109	0.276
OuterMembrane	2.072	0.405
Periplasmic	0.336	0.888
Cytoplasmic	0.224	2.951
InnerMembrane	0.240	0.480

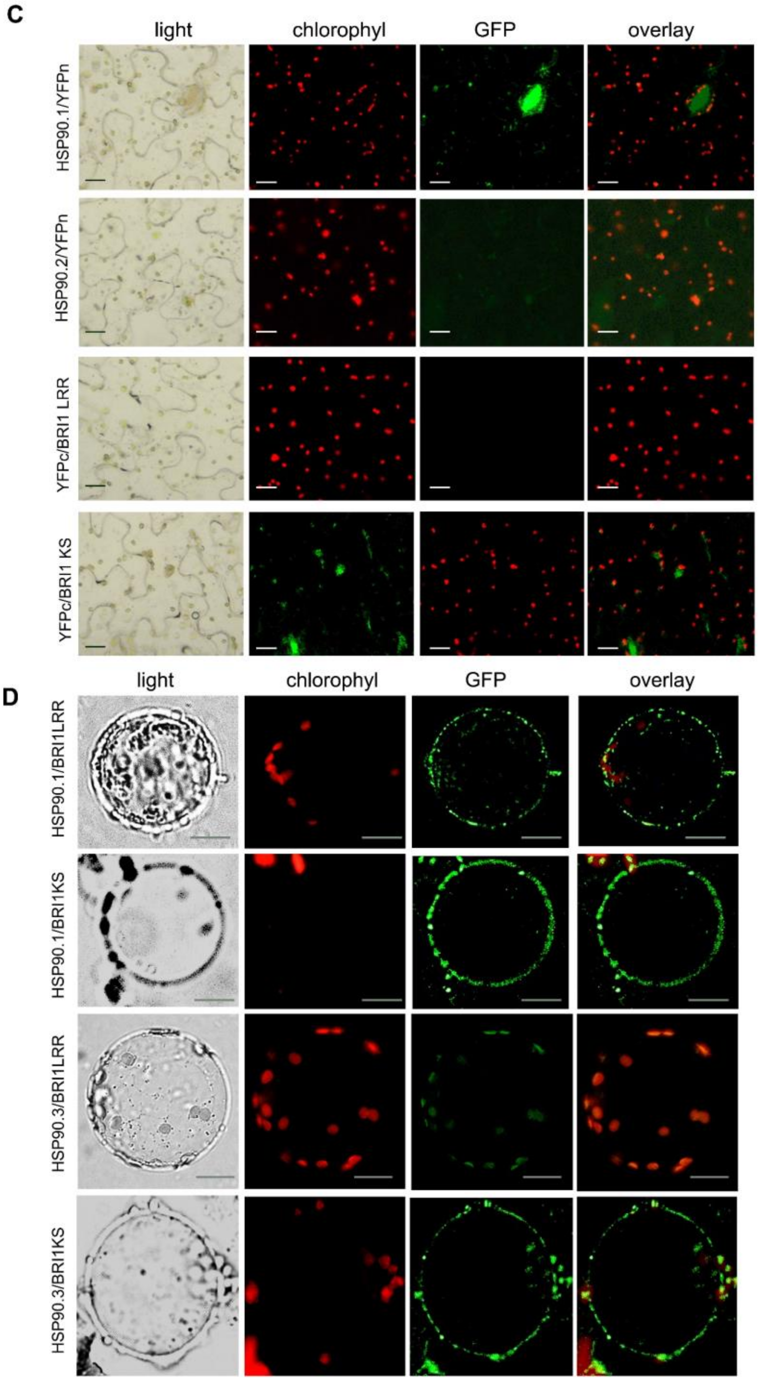

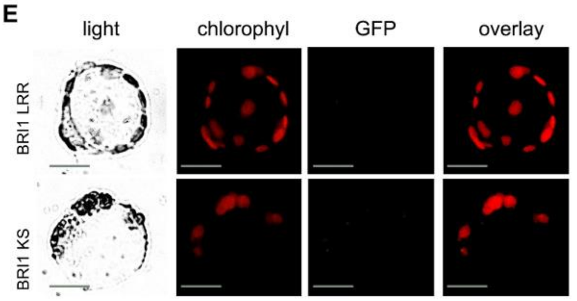

Supplementary Figure S3. BRI1LRR and BRI1 KS interact differently with HSP90.1 and HSP90.2. (**A**) Schematic depiction of BRI1 receptor domains. Two-headed arrows show BRI1, and its domains used for yeast 2-hybrid and BiFC interaction assays. For BRI1LRR amino acids (aa) 1-689 and for BRI1KS 642-1192 aa were used. LRR, Leucine-rich repeats (1-24). Diamond region and LRR21 (in pink) denote the BR ligand-binding site. TM, transmembrane domain; JM, juxtamembrane region; KD, kinase domain; AL, activation loop; CTD, C-terminal domain. Phosphorylated (P) aa are shown. (**B**) Subcellular localization predictions of BRI1LRR and BRI1KS receptor domains using the subcellular localization predictor algorithm (CELLO, http://cello.life.nctu.edu.tw). (**C**) Negative controls for HSP90.1 or HSP90.2 interactions with BRI1 LRR and BRI1 KS domains, respectively. (**D**) Interactions between HSP90 and BRI1LRR or BRI1KS domains by BiFC assays in tobacco protoplasts. Interaction was detected between HSP90.1 and BRI1LRR or BRI1KS, and between HSP90.2 and BRI1KS. (**E**) Controls for BRI1LRR and BRI1KS BiFC interactions. BRI1LRR pSPYNE co-expressed with pSPYCE vector (BRI1LRR), or BRI1KS pSPYNE co-expressed with pSPYCE vector (BRI1KS) in tobacco protoplasts. Scale bars: 20 μm.

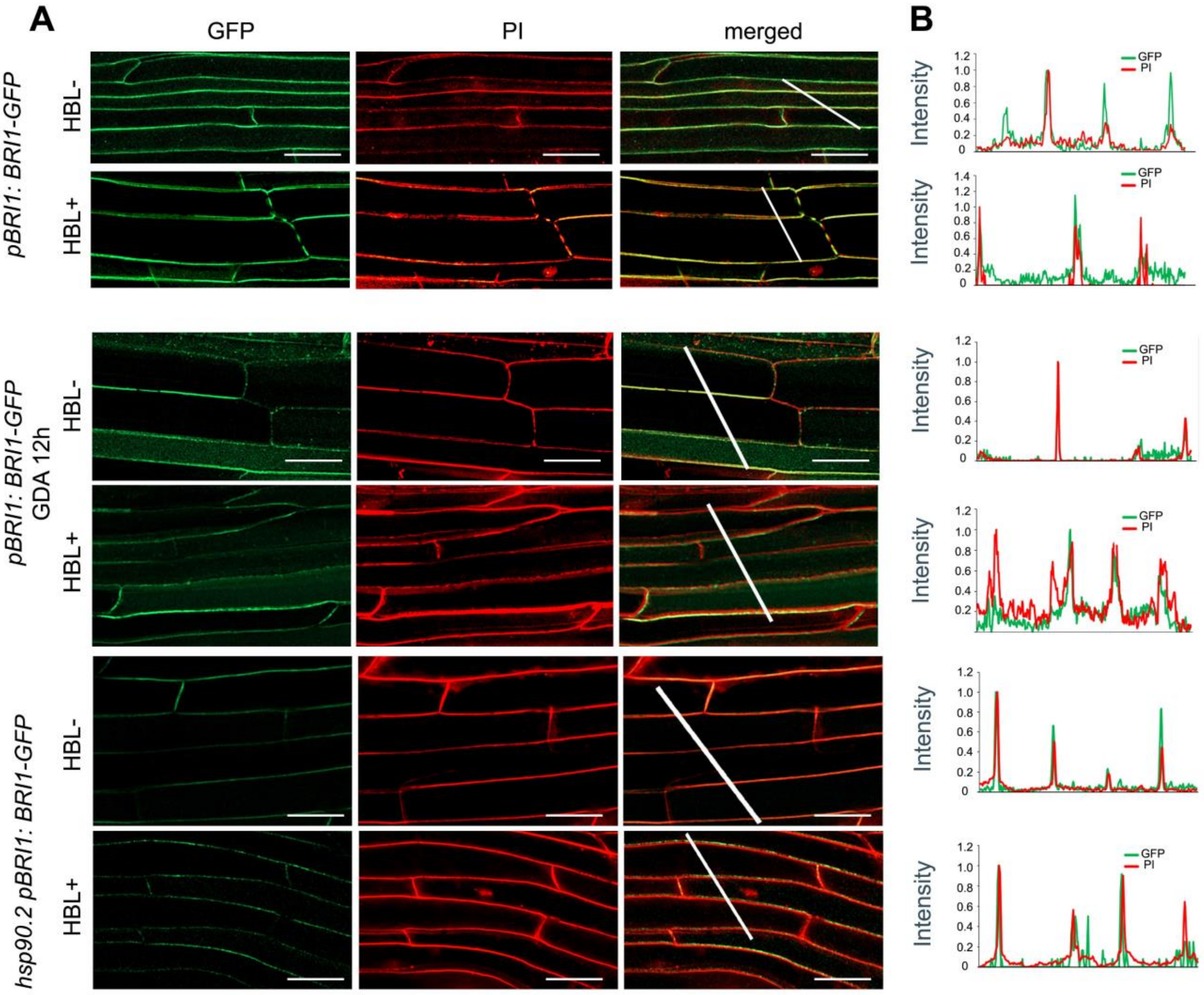

Supplementary Figure S4. BRI1 localization in hypocotyl cells of *hsp90.2*. (**A**-**B**) BRI1:BRI1-GFP localization in wild-type hypocotyl cells of 5-d-old etiolated seedlings growing under control conditions or after treatment with 10 µM GDA for 12 h or of *hsp90.2* mutant in resting conditions and upon the exogenous application of 100 nM HBL (A). The white lines mark the cells used for the quantification of the fluorescence signal intensity presented in plot profiles at the right of the images, generated by normalized values of fluorescence intensity. Propidium iodide (PI) cell-wall stain was used to visualize the cell patterns in etiolated hypocotyls (b). Scale bars, 20 µm.

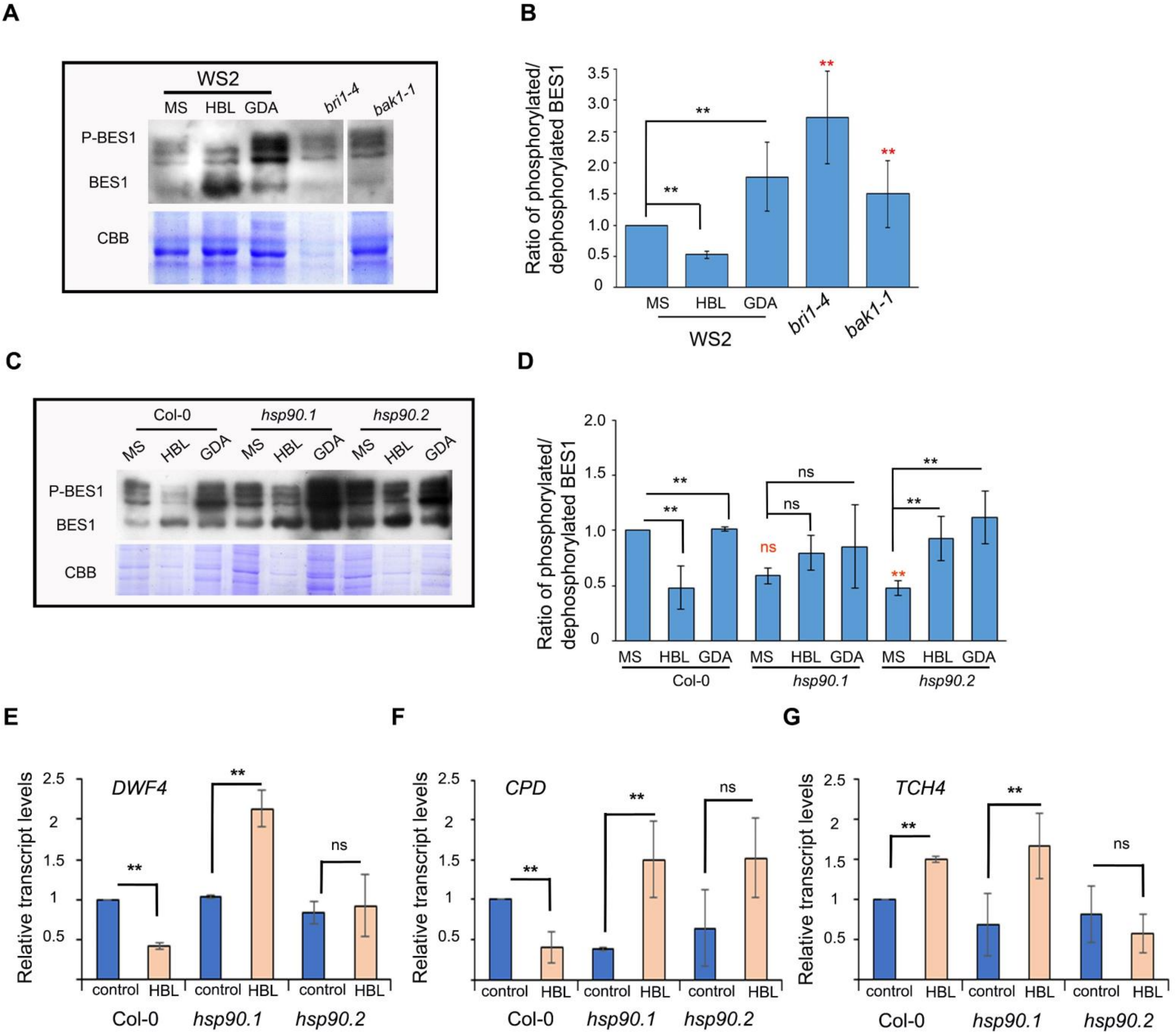

Supplementary Figure S5. HSP90 regulates the transcriptional activation of BES1. (**A**) Western blot analysis of BES1 in the indicated genotypes and treatments. Proteins were extracted from 5-day-old etiolated seedlings. (**B**) Quantification of the protein abundance of phosphorylated and non-phosphorylated forms of BES1 transcription factor in the indicated genotypes and treatments. Data are means and error bars indicate ± s.d. (standard deviation). Data were analyzed by t- test (******): P<0.01, ns: non-significant according to *t-test*. For the quantification at least 3 biological repetitions were used. (**C**) Western blot analysis of BES1 from proteins extracted from hypocotyls of 5-day-old etiolated seedlings of wild-type, *hsp90.1* and *hsp90.2* mutants grown under control conditions or in the presence of 60 nM HBL or 2 μM GDA. (**D**) Quantification of the ratio of phosphorylated to dephosphorylated BES1 protein levels in 5-day-old etiolated seedling wild-type and *hsp90.1* and *hsp90.2* mutants in the indicated treatments. Data are means and error bars indicate ±s.d. (standard deviation). Data were analyzed by One-way ANOVA followed by Tukey's test; *p<0.05, **p<0.01, ns – non-significant. The red stars in the diagrams show the statistically significant differences between wild-type and mutants grown under control conditions **(*):** P<0.05, **(**):** P<0.01, ns – non-significant. The experiment was repeated at least three times with similar results. (**E**-**G**) Relative transcript levels of the indicated genes in 7-day-old etiolated seedlings of wild type and *hsp90.1* and *hsp90.2* mutants under control conditions and after treatment with 60 nM HBL for 4 hours. *GAPDH* was used as a reference gene. Data are means and error bars indicate ±s.d. (standard deviation). **(*):** P<0.05, **(**):** P<0.01, ns – non-significant according to *t-test*.

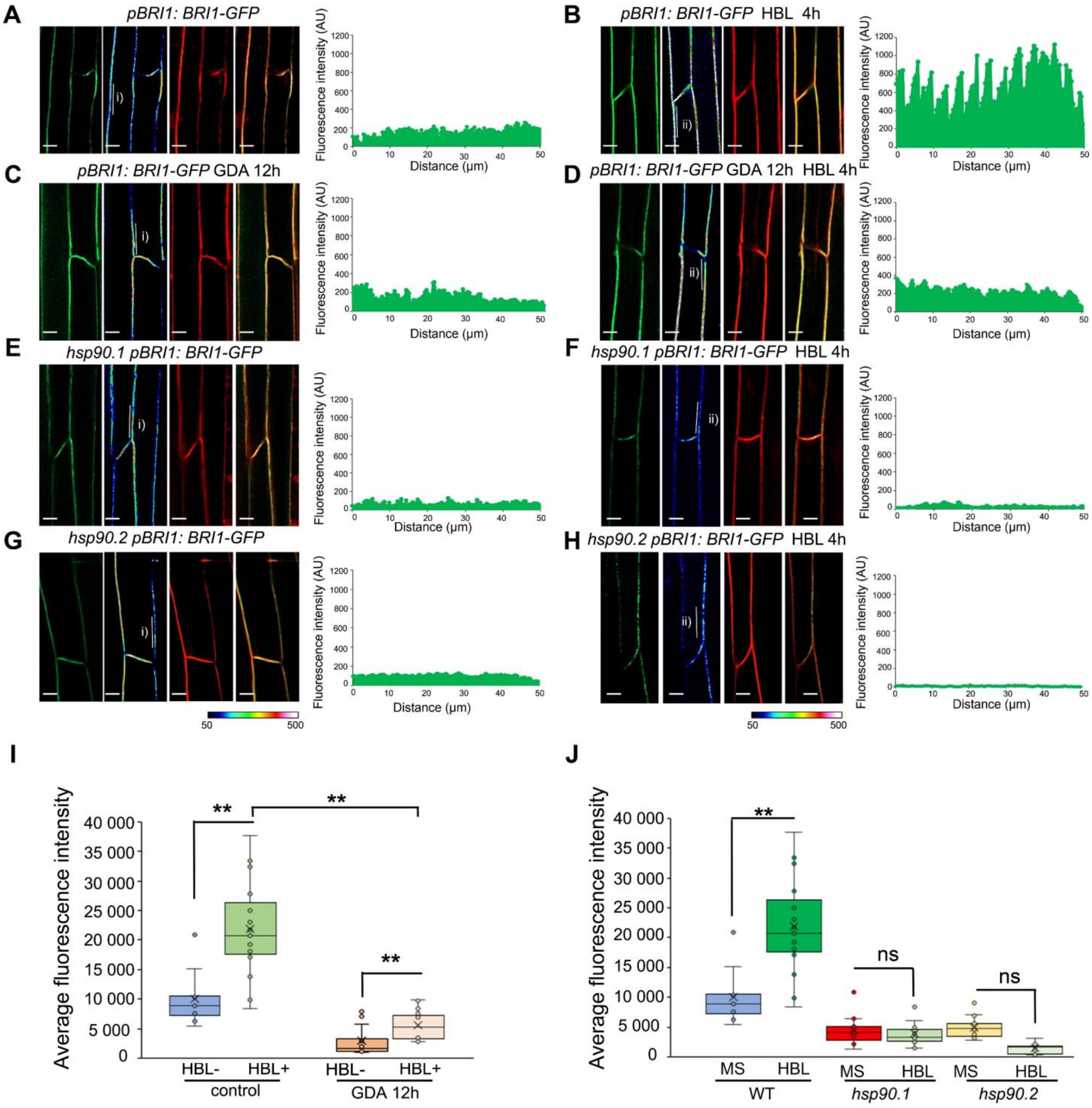

Supplementary Figure S6. Images used for the analysis of BRI1 spatial clustering index of wild-type and *hsp90* mutants after treatment with HBL and under control conditions. (**A-H**) Expression of BRI1 at the PM of etiolated hypocotyl cells in the indicated genotypes and treatments. From left to right: GFP fluorescent signals, semi-quantitative fluorescence intensity evaluation of BRI1-GFP expression levels using pseudo color-coded range from the scale, where dark blue represents zero intensity (50 in arbitrary units) and white represents maximum intensity (500 in arbitrary units), PI cell-wall stain used to visualize the cell patterns in etiolated hypocotyls, overlay of GFP and PI-stained images. Scale bars: 10 µm. (**I**) Quantification of the average fluorescence intensity of BRI1-GFP protein at hypocotyl cells of 5-day-old etiolated seedlings in the indicated treatments. (**J**) Quantification of the average fluorescence intensity of BRI1-GFP protein at hypocotyl cells of 5-day-old etiolated seedlings of the indicated genotypes untreated or treated with HBL (100 nM). In box plots the middle line in the box represents median, the × shows mean, the bottom line depicts the 1st quartile, while the top line describes the 3rd quartile; the vertical lines (whiskers) extend to the minimum and maximum value within the 1.5× interquartile range (distance between the 1st and the 3rd quartile); points outside of the whiskers mark outliers (values outside of the 1.5× interquartile range). The data were analysed with one-way ANOVA followed by Tukey's test, statistically significant differences compared to control are shown, ** is significant at $p<0.01$, ns- not significant.

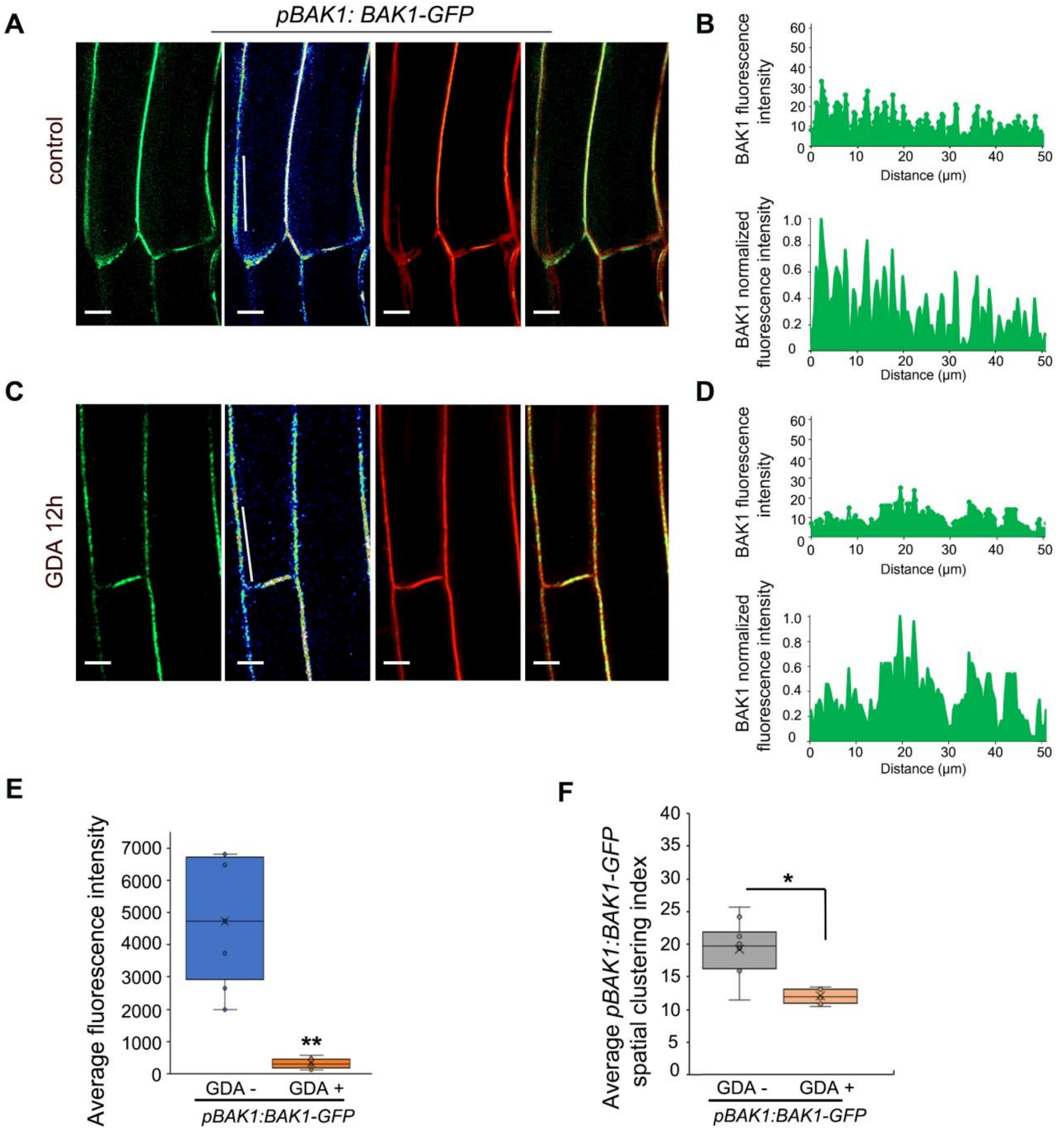

Supplementary Figure S7. HSP90 regulates the spatial distribution of BAK1 co-receptor at the PM. (**A, C**) Expression of BAK1 at the PM of etiolated hypocotyl cells in control conditions and upon treatment with 10 μM GDA for 12h. From left to right: GFP fluorescent signals, semi-quantitative fluorescence intensity evaluation of BRI1-GFP expression levels using pseudo color-coded range from the scale, where dark blue represents zero intensity (50 in arbitrary units) and white represents maximum intensity (500 in arbitrary units), PI cell-wall stain used to visualize the cell patterns in etiolated hypocotyls, overlay of GFP and PI-stained images. Scale bars: 10 μm. **(B, D**) BAK1 clustering index presented in plot profiles generated by the quantification of the fluorescence signal intensity of the white line marked PM regions of etiolated hypocotyls cells. Spatial clustering Index of BAK1 in etiolated hypocotyl cells in control conditions (B) and upon treatment with 10 μM GDA for 12h (D). **(E**) Comparison of the average spatial clustering index of BRI1 and BAK1 at the PM of etiolated hypocotyl cells of treated and untreated seedlings with 10 μM GDA for 12h. In box plots the middle line in the box represents median, the × shows mean, the bottom line depicts the 1st quartile, while the top line describes the 3rd quartile; the vertical lines (whiskers) extend to the minimum and maximum value within the 1.5× interquartile range (distance between the 1st and the 3rd quartile); points outside of the whiskers mark outliers (values outside of the 1.5× interquartile range). The data were analysed with One-way ANOVA followed by Tukey's test, statistically significant differences compared to control are shown, ** is significant at $p<0.01$, ns- not significant.

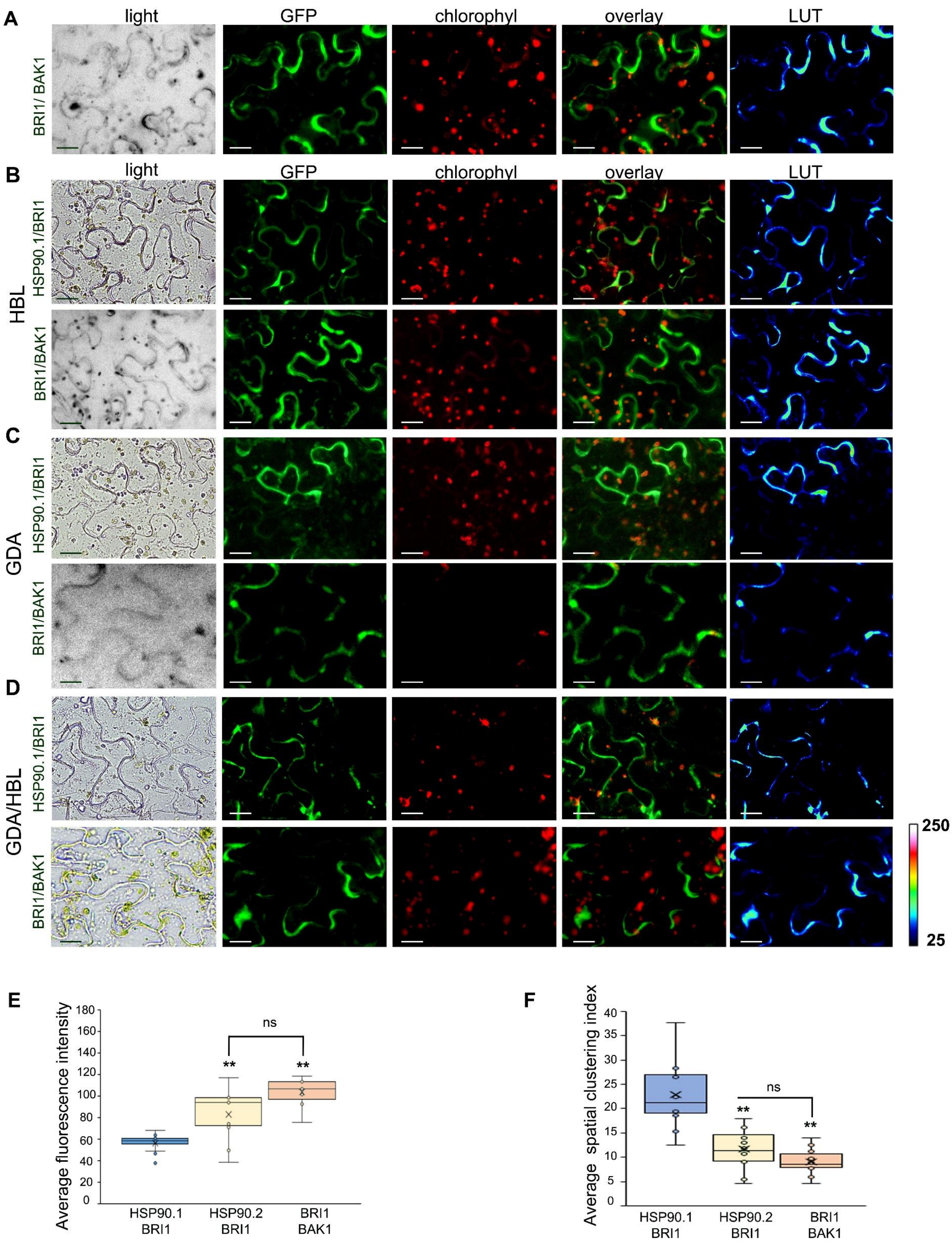

A
B
C
D
E
F
light
GFP
chlorophyl
overlay
LUT
BRI1/ BAK1
HBL
HSP90.1/BRI1
BRI1/BAK1
GDA
GDA/HBL
250
25
Average fluorescence intensity
180
160
140
120
100
80
60
40
20
0
ns
**
HSP90.1 BRI1
HSP90.2 BRI1
BRI1 BAK1
Average spatial clustering index
40
35
30
25
20
15
10
5
0

Supplementary Figure S8. (**A-D**) Images of BiFC interaction assays of HSP90.1 with full length BRI1 protein or BRI1 with BAK1 in resting conditions (A), after treatment with 60 nM HBL for 4h (B), or in the presence of GDA 10 µM for 12h (C) or in the presence of 60 nM HBL for 4 hours after treatment with 10 µM GDA for 12h (D). From left to right: fluorescent transparent light images, YFP signals, chlorophyl autofluorescence, and semi-quantitative fluorescence intensity evaluation of HSP90.1/BRI1 or BRI1/BAK1 levels using pseudo color-coded range from the scale, where dark blue represents zero intensity (25 in arbitrary units) and white represents maximum intensity (250 in arbitrary units). Scale bars, 20 µm. (**E**) Schematic representation of the quantification of the relative spatial plasma membrane distribution of the fluorescent signal in *N. benthamianna* leaf epidermal cells. (**F**) Quantification of the distribution at the PM of HSP90.1/BRI1 and BRI1/BAK1 complexes at the indicated conditions. Box plots show the first and third quartiles, split by the median (line) and mean (cross). Data are analyzed by one-way ANOVA followed by Holm's test; *$p<0.05$, **$p<0.01$. The red stars in the diagram show the statistically significant differences between the treatments analyzed by two-way ANOVA.

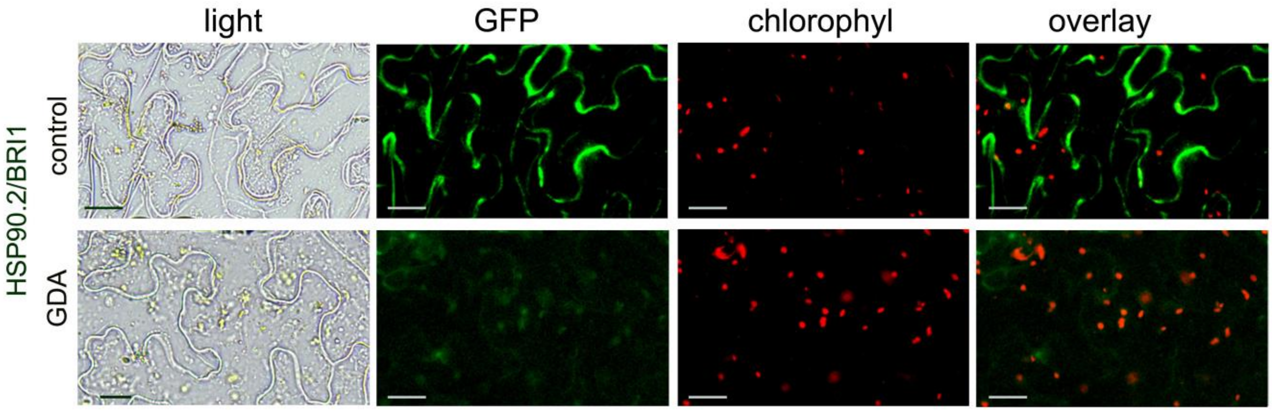

Supplementary Figure S9. **HSP90.2 does not interact with BRI1 receptor in the presence of GDA. (A)** BiFC assays for the interactions of HSP90.1 and HSP90.2 with BRI1 receptor or BAK1 with BRI1. **(B)** Treatment with GDA 2μM for 12h abolishes the interactions of HSP90.2 with BRI1 receptor. Scale bars: 20 μm.

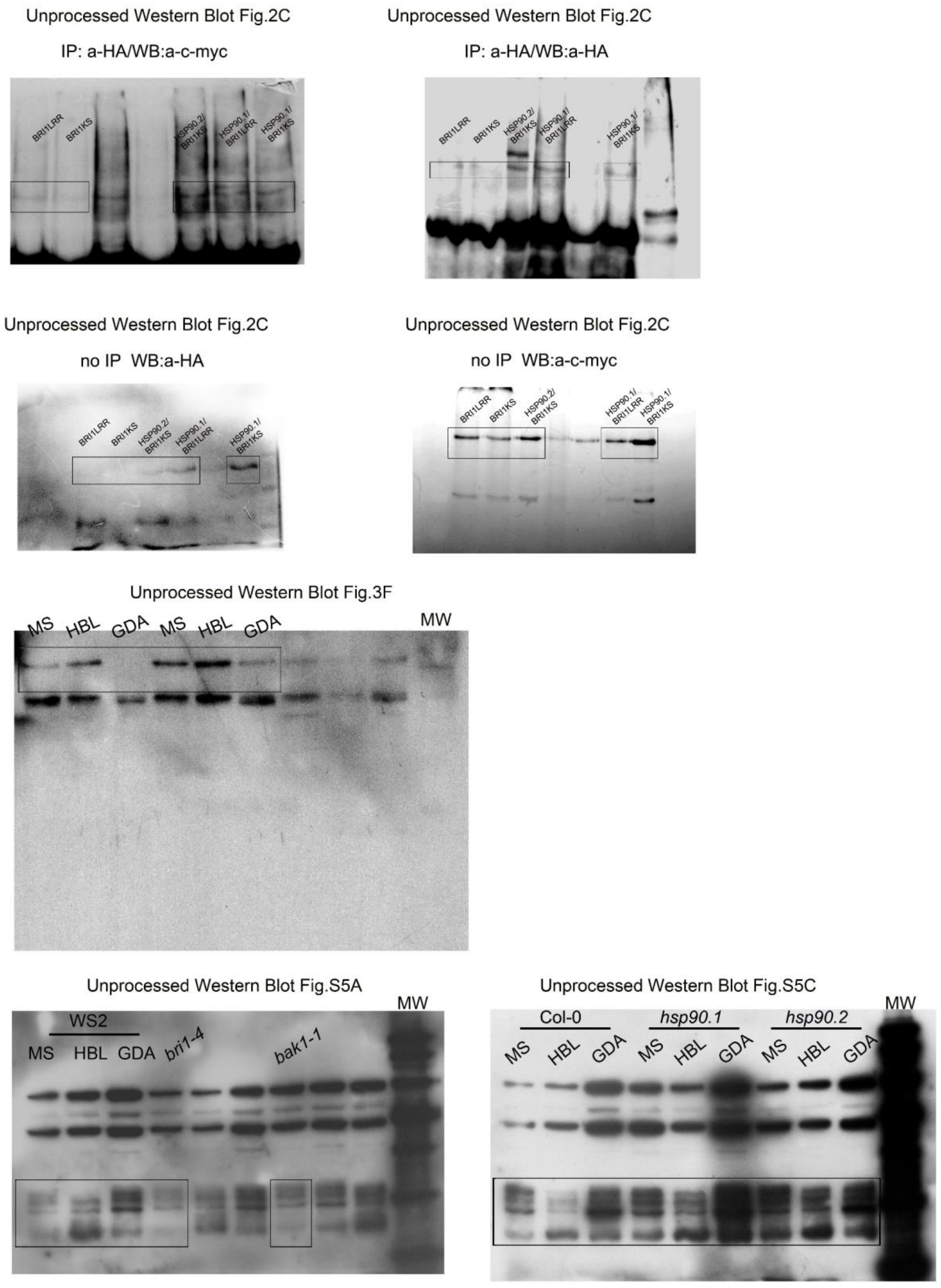

Supplementary Figure S10. Unprocessed Western blot images used in this study.

Supplementary Table S1. Primers List

Primers for yeast two hybrid interactions		
Gene	Primer	Sequence
At5g52640	Hsp90.1yeastFor	5’-AAAGGATCCCGATGGCGGATGTTCAG-3´
	Hsp90.1yeastRev	5’–TTTCTCGAGGTCGACTTCCTCCATCTTGC-3´,
At5g56030	Hsp90.2yeastFor	5´-ATACATATGATGGCGGACGCTGAAACC-3´
	Hsp90.2yeastRev	5´-ATAATCGATTTAGTCGACTTTTGTGTG-3´
At4g39400	BRI1LRRyeastFor	5´-GGCCCATATGAGCTTCACCATCTCAG-3´
	BRI1LRRyeastRev	5´-TGGGGGATCCTTCCGTAAGCATAGTAAGAGCT-3´
	BRI1KSyeastFor	5´-CAAGCATATGGGGAACATTCCGGCTGAGC-3´
	BRI1KSyeastRev	5´-ACCCCGGATCCAGGAACTTCTTTTATACTCATATCAACC-3´
At4g33430	BAK1 yeastFor	5´-TGAATTCATGGAACGAAGATTAATGATC-3´
	BAK1 yeastRev	5´-TTTTCTCGAGTCTTGGACCCGAGGGGTATTCG-3´
	BAK1LRRyeastFor	5´-TGAATTCATGGAACGAAGATTAATGATC-3´
	BAK1LRRyeastRev	5´-ATAGTCGACCCACCAAGCTAGTGCAATGG-3´
	BAK1KSyeastFor	5´-TGAATTCCGAAGGAAAAAGCCGCAGGAC-3´
	BAK1KSyeastRev	5´-TTTTCTCGAGTCTTGGACCCGAGGGGTATTCG-3´
Primers for BiFC contructs		
Gene	Primer	Sequence
At5g52640	Hsp90.1splitFor	5´-TTAATGGATCCAAGTTCGTTGCGATGGCGGATG-3´
	Hsp90.1splitRev	5´- TTTCTCGAGGTCGACTTCCTCCATCTTGC-3´
At5g56030	Hsp90.2splitFor	5´-ATATCTAGAACGACAATGGCGGACGCTGAAACC-3´
	Hsp90.2splitRev	5´-ATAGGTACCGTCGACTTCCTCCATCTT-3´
At4g39400	BRI1 For	5'-GGATCCATGAAGACTTTTTCAAGC-3'
	BRI1 Rev	5’-CCCCGGTACCAGGAACTTCTTTTATACTCATATCAACC-3’
	BRI1LRRsplitFor	5’-GGATCCATGAAGACTTTTTCAAGC-3’
	BRI1LRRsplitRev	5´-GCCTGGTACCTTCCGTAAGCATAGTAAGAGCT-3´
	BRI1KSsplitFor	5´-AAAAGGATCCGGGAACATTCCGGCTGAGC-3´
	BRI1KSsplitRev	5´-CCCCGGTACCAGGAACTTCTTTTATACTCATATCAACC-3´
At4g33430	BAK1splitFor	5’-CCCTGGATCCATGGAACGAAGATTAATGATCC – 3’
	BAK1splitRev	5’–TTTTCTCGAGTCTTGGACCCGAGGGGTATT G – 3’
Primers for Quantitative RT PCR		
Gene	Primer	Sequence
oligo -dT primer		5´-GTCGACCTCGAGTTTTTTTTTTTTTTTTT-3´
At3g04120	GAPDHF	5’-GGCCTTCAATGAAGGACTGGAGAG-3’
	GAPDHR	5’-GCTCGACCTGTTGTCGCCAACG-3’
18S	18SF	5´-TTGATTCTATGGGTGGTGGT-3´
	18SR	5´-CCTTGTTACGACTTCTCCTT-3´
At5g57560	TCH4F	5´-GGAACAGTCACAACACTTTACTT-3´
	TCH4R	5´-ACAACCACGAGCCAGTAGTA-3´
At3g50660	DWF4-F	5´-CAAGAAGGAACTAGGAGAGTCAG-3´
	DWF4-R	5´-CACGTCGAAAAACTACCACTT-3´
A5g05690	CPD-F	5´-TGAATGGAGTGATTACAAGTCA-3´
	CPD-R	5´-GAACACATTAGAAGGGCCTG-3´

Supplementary Table S2. Statistical analysis of Fig.1A.

treatments	Tukey HSD	Tukey HSD	Tukey HSD
pair	Q statistic	p-value	inferfence
WT MS vs WT BL	64.945	0.0010053	** p<0.01
WT MS vs WT GDA	157.864	0.0010053	** p<0.01
WT MS vs *hsp90.1*	124.902	0.0010053	** p<0.01
WT MS vs *hsp90.1* BL	141.393	0.0010053	** p<0.01
WT MS vs *hsp90.1* GDA	207.331	0.0010053	** p<0.01
WT MS vs *hsp90.2*	67.630	0.0010053	** p<0.01
WT MS vs *hsp90.2* BL	131.454	0.0010053	** p<0.01
WT MS vs *hsp90.2* GDA	117.067	0.0010053	** p<0.01
WT BL vs WT GDA	109.110	0.0010053	** p<0.01
WT BL vs *hsp90.1*	66.616	0.0010053	** p<0.01
WT BL vs *hsp90.1* BL	85.949	0.0010053	** p<0.01
WT BL vs *hsp90.1* GDA	165.600	0.0010053	** p<0.01
WT BL vs *hsp90.2*	0.1907	0.8999947	insignificant
WT BL vs *hsp90.2* BL	78.507	0.0010053	** p<0.01
WT BL vs *hsp90.2* GDA	63.469	0.0010053	** p<0.01
WT GDA vs *hsp90.1*	54.098	0.0049464	** p<0.01
WT GDA vs *hsp90.1* BL	36.818	0.1893779	insignificant
WT GDA vs *hsp90.1* BL	46.703	0.0293281	* p<0.05
WT GDA vs *hsp90.2* BL	120.527	0.0010053	** p<0.01
WT GDA vs *hsp90.2* BL	29.854	0.4698901	insignificant
WT GDA vs *hsp90.2* GDA	40.226	0.1072051	insignificant
hsp90.1 vs *hsp90.1* BL	20.676	0.8617929	insignificant
hsp90.1 vs *hsp90.1* GDA	112.214	0.0010053	** p<0.01
hsp90.1 vs *hsp90.2*	76.363	0.0010053	** p<0.01
hsp90.1 vs *hsp90.2* BL	20.811	0.8560624	insignificant
hsp90.1 vs *hsp90.2* GDA	0.7057	0.8999947	insignificant
hsp90.1 BL vs *hsp90.1* GDA	94.163	0.0010053	** p<0.01
hsp90.1 BL vs *hsp90.2*	98.235	0.0010053	** p<0.01
hsp90.1 BL vs *hsp90.2* BL	0.3138	0.8999947	insignificant
hsp90.1 BL vs *hsp90.2* GDA	0.9905	0.8999947	insignificant

hsp90.1 GDA vs *hsp90.2*	184.849	0.0010053	** p<0.01
hsp90.1 GDA vs *hsp90.2* BL	78.739	0.0010053	** p<0.01
hsp90.1 GDA vs *hsp90.2* GDA	87.529	0.0010053	** p<0.01
hsp90.2 vs *hsp90.2* BL	87.240	0.0010053	** p<0.01
hsp90.2 vs *hsp90.2* GDA	70.292	0.0010053	** p<0.01
hsp90.2 BL vs *hsp90.2* GDA	11.544	0.8999947	insignificant

Supplementary Table S3. Statistical analysis of Fig.1C (Col-0).

treatments	Tukey HSD	Tukey HSD	Tukey HSD
pair	Q statistic	p-value	inferfence
0 nM BL vs 0,1 nM BL	4,3402	0,0381831	* p<0.05
0 nM BL vs 1 nM BL	0,5491	0,8999947	insignificant
0 nM BL vs 10 nM BL	2,584	0,5265037	insignificant
0 nM BL vs 60 nM BL	5,3113	0,0040458	** p<0.01
0 nM BL vs 100 nM BL	7,6884	0,0010053	** p<0.01
0 nM BL vs 1000 nM BL	13,7778	0,0010053	** p<0.01
0,1 nM vs 1 nM BL	3,1822	0,2731574	insignificant
0,1 nM vs 10 nM BL	7,3332	0,0010053	** p<0.01
0,1 nM vs 60 nM BL	9,6899	0,0010053	** p<0.01
0,1 nM vs 100 nM BL	12,3957	0,0010053	** p<0.01
0,1 nM vs 1000 nM BL	18,1471	0,0010053	** p<0.01
1 nM BL vs 10 nM BL	2,8054	0,4300972	insignificant
1 nM BL vs 60 nM BL	5,229	0,0049854	** p<0.01
1 nM BL vs 100 nM BL	7,2889	0,0010053	** p<0.01
1 nM BL vs 1000 nM BL	12,875	0,0010053	** p<0.01
1 nM BL vs 60 nM BL	3,1097	0,300162	insignificant
10 nM BL vs 100 nM BL	5,5242	0,0023261	** p<0.01
10 nM BL vs 1000 nM BL	12,0746	0,0010053	** p<0.01
60 nM BL vs 100 nM BL	2,0678	0,7393731	insignificant
60 nM BL vs 1000 nM BL	8,5249	0,0010053	** p<0.01
100 nM BL vs 1000 nM BL	6,873	0,0010053	** p<0.01

Supplementary Table S4. Statistical analysis of Fig.1C (*hsp90.1*).

treatments	Tukey HSD	Tukey HSD	Tukey HSD
pair	Q statistic	p-value	inferfence
0 nM BL vs 0,1 nM BL	104.370	0.0010053	** p<0.01
0 nM BL vs 1 nM BL	169.700	0.0010053	** p<0.01
0 nM BL vs 10 nM BL	189.378	0.0010053	** p<0.01
0 nM BL vs 60 nM BL	29.807	0.3513103	insignificant
0 nM BL vs 100 nM BL	34.352	0.1899788	insignificant
0 nM BL vs 1000 nM BL	50.161	0.0081427	** p<0.01
0,1 nM vs 1 nM BL	51.977	0.0051847	** p<0.01
0,1 nM vs 10 nM BL	66.977	0.0010053	** p<0.01
0,1 nM vs 60 nM BL	80.561	0.0010053	** p<0.01
0,1 nM vs 100 nM BL	73.333	0.0010053	** p<0.01
0,1 nM vs 1000 nM BL	153.298	0.0010053	** p<0.01
1 nM BL vs 10 nM BL	15.085	0.8999947	insignificant
1 nM BL vs 60 nM BL	146.894	0.0010053	** p<0.01
1 nM BL vs 100 nM BL	136.642	0.0010053	** p<0.01
1 nM BL vs 1000 nM BL	225.160	0.0010053	** p<0.01
1 nM BL vs 60 nM BL	167.040	0.0010053	** p<0.01
10 nM BL vs 100 nM BL	155.650	0.0010053	** p<0.01
10 nM BL vs 1000 nM BL	247.175	0.0010053	** p<0.01
60 nM BL vs 100 nM BL	0.5693	0.8999947	insignificant
60 nM BL vs 1000 nM BL	83.007	0.0010053	** p<0.01
100 nM BL vs 1000 nM BL	85.944	0.0010053	** p<0.01

Supplementary Table S5. Statistical analysis of Fig.1C (*hsp90.2*).

treatments	Tukey HSD	Tukey HSD	Tukey HSD
pair	Q statistic	p-value	inferfence
0 nM BL vs 0,1 nM BL	39.232	0.0851639	insignificant
0 nM BL vs 1 nM BL	111.186	0.0010053	** p<0.01
0 nM BL vs 10 nM BL	75.025	0.0010053	** p<0.01
0 nM BL vs 60 nM BL	13.452	0.8999947	insignificant
0 nM BL vs 100 nM BL	0.7454	0.8999947	insignificant
0 nM BL vs 1000 nM BL	59.193	0.0010053	** p<0.01
0,1 nM vs 1 nM BL	69.182	0.0010053	** p<0.01
0,1 nM vs 10 nM BL	32.650	0.2435583	insignificant
0,1 nM vs 60 nM BL	23.161	0.6369207	insignificant
0,1 nM vs 100 nM BL	32.824	0.2377046	insignificant
0,1 nM vs 1000 nM BL	98.966	0.0010053	** p<0.01
1 nM BL vs 10 nM BL	40.262	0.0704281	insignificant
1 nM BL vs 60 nM BL	88.531	0.0010053	** p<0.01
1 nM BL vs 100 nM BL	106.593	0.0010053	** p<0.01
1 nM BL vs 1000 nM BL	176.455	0.0010053	** p<0.01
1 nM BL vs 60 nM BL	54.900	0.0024871	** p<0.01
10 nM BL vs 100 nM BL	69.287	0.0010053	** p<0.01
10 nM BL vs 1000 nM BL	140.321	0.0010053	** p<0.01
60 nM BL vs 100 nM BL	0.6830	0.8999947	insignificant
60 nM BL vs 1000 nM BL	68.757	0.0010053	** p<0.01
100 nM BL vs 1000 nM BL	68.380	0.0010053	** p<0.01

Supplementary Table S6. Statistical analysis of Fig.3B.

treatments	Tukey HSD	Tukey HSD	Tukey HSD
pair	Q statistic	p-value	inferfence
control vs BL 4h	81.277	0.0010053	** p<0.01
control vs GDA 12h	129.734	0.0010053	** p<0.01
control vs GDA12h BL 4h	136.226	0.0010053	** p<0.01
control vs GDA 24h	160.354	0.0010053	** p<0.01
control vs GDA 24h BL 4h	166.327	0.0010053	** p<0.01
BL 4h vs GDA 12h	226.284	0.0010053	** p<0.01
BL 4h vs GDA 12h BL 4h	235.198	0.0010053	** p<0.01
BL 4h vs GDA 24h	261.135	0.0010053	** p<0.01
BL 4h vs GDA 24h BL 4h	265.527	0.0010053	** p<0.01
GDA 12h vs GDA 12h BL 4h	0.4762	0.8999947	insignificant
GDA 12h vs GDA 24h	30.699	0.2643688	insignificant
GDA 12h vs GDA 24h BL 4h	39.242	0.0736611	insignificant
GDA12h BL 4h vs GDA 24h	26.431	0.4314414	insignificant
GDA 12h vs GDA 24h	35.201	0.1410890	insignificant
GDA 24h vs GDA 24h BL 4h	0.9264	0.8999947	insignificant

Supplementary Table S7. Statistical analysis of Fig.3C.

treatments<br><br>pair	Tukey HSD<br><br>Q statistic	Tukey HSD<br><br>p-value	Tukey HSD<br><br>inferfence
WT control vs BL 4h	111.242	0.0010053	** p<0.01
WT control vs *hsp90.1*	198.547	0.0010053	** p<0.01
WT control vs *hsp90.1* BL 4h	227.361	0.0010053	** p<0.01
WT control vs *hsp90.2*	211.006	0.0010053	** p<0.01
WT control vs *hsp90.2* BL 4h	263.662	0.0010053	** p<0.01
BL 4h vs *hsp90.1*	334.914	0.0010053	** p<0.01
BL 4h vs *hsp90.1* BL 4h	370.769	0.0010053	** p<0.01
BL 4h vs *hsp90.2*	345.572	0.0010053	** p<0.01
BL 4h vs *hsp90.2* BL 4h	417.969	0.0010053	** p<0.01
hsp90.1 vs *hsp90.1* BL 4h	25.811	0.4573951	insignificant
hsp90.1 vs *hsp90.2*	16.999	0.8136746	insignificant
hsp90.1 vs *hsp90.2* BL 4h	57.135	0.0016507	** p<0.01
hsp90.1 BL 4h vs *hsp90.2*	0.7762	0.8999947	insignificant
hsp90.1 BL 4h vs *hsp90.2* BL 4h	31.197	0.2464771	insignificant
hsp90.2 vs *hsp90.2* BL 4h	37.506	0.0967486	insignificant

Supplementary Table S8. Statistical analysis of Fig.3E.

treatments pair	Tukey HSD Q statistic	Tukey HSD p-value	Tukey HSD inferfence
WT vs WT BL	352.244	0.0010053	** p<0.01
WTvs WT GDA	144.382	0.0010053	** p<0.01
WT vs *bri1-4*	412.147	0.0010053	** p<0.01
WT vs *bri1-4* BL	379.125	0.0010053	** p<0.01
WT vs *bri1-4* GDA	344.985	0.0010053	** p<0.01
WT vs *bak1-1*	283.494	0.0010053	** p<0.01
WT vs *bak1-1* BL	274.625	0.0010053	** p<0.01
WT vs *bak1-1* GDA	464.113	0.0010053	** p<0.01
WT BL vs WT GDA	228.325	0.0010053	** p<0.01
WT BL vs *bri1-4*	97.493	0.0010053	** p<0.01
WT BL vs *bri1-4* BL	63.783	0.0010053	** p<0.01
WT BL vs *bri1-4* GDA	70.455	0.0010053	** p<0.01
WT BL vs *bak1-1*	113.406	0.0010053	** p<0.01
WT BL vs *bak1-1* BL	83.649	0.0010053	** p<0.01
WT BL vs *bak1-1* GDA	94.339	0.0010053	** p<0.01
WT GDA vs *bri1-4*	303.238	0.0010053	** p<0.01
WT GDA vs *bri1-4* BL	268.395	0.0010053	** p<0.01
WT GDA vs *bri1-4* GDA	246.072	0.0010053	** p<0.01
WT GDA vs *bak1-1*	139.992	0.0010053	** p<0.01
WT GDA vs *bak1-1* BL	143.900	0.0010053	** p<0.01
WT GDA vs *bak1-1* GDA	342.396	0.0010053	** p<0.01
bri1-4 vs *bri1-4* BL	30.773	0.4288640	insignificant
bri1-4 vs *bri1-4* GDA	13.033	0.8999947	insignificant
bri1-4 vs *bak1-1*	205.663	0.0010053	** p<0.01
bri1-4 vs *bak1-1* BL	173.036	0.0010053	** p<0.01
bri1-4 vs *bak1-1* GDA	18.794	0.8999947	insignificant
bri1-4 BL vs *bri1-4* GDA	14.190	0.8999947	insignificant
bri1-4 BL vs *bak1-1*	168.736	0.0010053	** p<0.01
bri1-4 BL vs *bak1-1* BL	139.015	0.0010053	** p<0.01
bri1-4 BL vs *bak1-1* GDA	16.522	0.8999947	insignificant

bri1-4 GDA vs *bak1-1*	158.876	0.0010053	** p<0.01
bri1-4 GDA vs *bak1-1* BL	135.435	0.0010053	** p<0.01
bri1-4 GDA vs *bak1-1* GDA	0.1615	0.8999947	insignificant
bak1-1 vs *bak1-1* BL	20.768	0.8569128	insignificant
bak1-1 vs *bak1-1* GDA	230.129	0.0010053	** p<0.01
bak1-1 BL vs *bak1-1* GDA	184.420	0.0010053	** p<0.01

Supplementary Table S9. Statistical analysis of Fig.4E.

treatments pair	Tukey HSD Q statistic	Tukey HSD p-value	Tukey HSD inferfence
control vs GDA 12h	0.4408	0.8999947	insignificant
control vs GDA 12h/BL4h	37.315	0.0335552	* p<0.05
GDA 12h vs GDA 12h/BL 4h	41.817	0.0157944	* p<0.05

Supplementary Table S10. Statistical analysis of Fig.5E.

treatments	Tukey HSD	Tukey HSD	Tukey HSD
pair	Q statistic	p-value	inferfence
control (BL-) vs GDA	82609	0.0010053	** p<0.01
control (BL-) vs control (BL+)	79470	0.0010053	** p<0.01
control (BL-) vs GDA (BL+)	30358	0.1536069	insignificant
GDA vs control (BL+)	0.2936	0.8999947	insignificant
GDA vs GDA (BL+)	47090	0.0089044	** p<0.01
control (BL+) vs GDA (BL+)	44218	0.0155037	* p<0.05

Supplementary Table S11. Statistical analysis of Fig.5F.

treatments	Tukey HSD	Tukey HSD	Tukey HSD
pair	Q statistic	p-value	inferfence
WT (BL-) vs WT (BL+)	58921	0.0010367	** p<0.01
WT (BL-) vs *hsp90.1* (BL-)	69103	0.0010053	** p<0.01
WT (BL-) vs *hsp90.1* (BL+)	42652	0.0385434	* p<0.05
WT (BL-) vs *hsp90.2* (BL-)	43706	0.0045958	** p<0.01
WT (BL-) vs *hsp90.2* (BL+)	31104	0.2490905	insignificant
WT (BL+) vs *hsp90.1* (BL-)	0.7481	0.8999947	insignificant
WT (BL+) vs *hsp90.1* (BL+)	18535	0.7519976	insignificant
WT (BL+) vs *hsp90.2* (BL-)	29810	0.2935264	insignificant
WT (BL+) vs *hsp90.2* (BL+)	29470	0.3058563	insignificant
hsp90.1 (BL-) vs *hsp90.1* (BL+)	27144	0.3994750	insignificant
hsp90.1 (BL-) vs *hsp90.2* (BL-)	38337	0.0840582	insignificant
hsp90.1 (BL-) vs *hsp90.2* (BL+)	38505	0.0816998	insignificant
hsp90.1 (BL+) vs *hsp90.2* (BL-)	12779	0.8999947	insignificant
hsp90.1 (BL+) vs *hsp90.2* (BL+)	11549	0.8999947	insignificant
hsp90.2 (BL-) vs *hsp90.2* (BL+)	0.1844	0.8999947	insignificant

Supplementary Table S12. Statistical analysis of Fig.6E.

Treatment pairs	Tukey HSD Q statistic	Tukey HSD p-value	Tukey HSD inferfence
HSP90.1/BRI1 control vs HSP90.1/BRI1 BL	72406	0.0010053	** p<0.01
HSP90.1/BRI1 control vs HSP90.1/BRI1 GDA	117371	0.0010053	** p<0.01
HSP90.1/BRI1 control vs HSP90.1/BRI1 GDA/BL	74926	0.0010053	** p<0.01
HSP90.1/BRI1 BL vs HSP90.1/BRI1 GDA	192101	0.0010053	** p<0.01
HSP90.1/BRI1 BL vs HSP90.1/BRI1 GDA/BL	113502	0.0010053	** p<0.01
HSP90.1/BRI1 GDA vs HSP90.1/BRI1 GDA/BL	12188	0.8040670	insignificant

Supplementary Table S13. Statistical analysis of Fig.6F.

treatments	Tukey HSD	Tukey HSD	Tukey HSD
pair	Q statistic	p-value	inferfence
BRI1/BAK1 control vs BRI1BAK1 BL	44501	0.0171936	* p<0.05
BRI1/BAK1 control vs BRI1BAK1 GDA	148363	0.0010053	** p<0.01
BRI1/BAK1 control vs BRI1/BAK1 GDA/BL	62650	0.0010053	** p<0.01
BRI1/BAK1 BL vs BRI1/BAK1 GDA	180648	0.0010053	** p<0.01
BRI1/BAK1 BL vs BRI1/BAK1 GDA/BL	99577	0.0010053	** p<0.01
BRI1/BAK1 GDA vs BRI1/BAK1 GDA/BL	73498	0.0010053	** p<0.01

Supplementary Table S14. Statistical analysis of Fig.6G.

Treatment pairs	Tukey HSD<br><br>Q statistic	Tukey HSD<br><br>p-value	Tukey HSD<br><br>inferfence
HSP90.1/BRI1 control vs BRI1/BAK1 control	123485	0.0010053	** p<0.01
HSP90.1/ BRI1control vs HSP90.1/BRI1 BL	108050	0.0010053	** p<0.01
HSP90.1/ BRI1 control vs BRI1/BAK1 BL	81008	0.0010053	** p<0.01
HSP90.1/BRI1 control vs HSP90.1/BRI1 GDA	31130	0.3617370	insignificant
HSP90.1 BRI1 control vs BRI1/BAK1 GDA	91176	0.0010053	** p<0.01
HSP90.1 BRI1 control vs HSP90.1/BRI1 GDA/BL	55018	0.0045081	** p<0.01
HSP90.1 BRI1 control vs BRI1/BAK1 GDA/BL	33432	0.2716312	insignificant
BRI1/BAK1 control vs HSP90.1/BRI1 BL	20096	0.8271143	insignificant
BRI1/BAK1 control vs BRI1/BAK1 BL	49156	0.0170647	* p<0.05
BRI1/BAK1 control vs HSP90.1/BRI1 GDA	86608	0.0010053	** p<0.01
BRI1/BAK1 control vs BRI1/BAK1 GDA	40835	0.0873235	insignificant
BRI1/BAK1 control vs HSP90.1/BRI1 GDA/BL	59307	0.0015732	** p<0.01
BRI1/BAK1 control vs BRI1/BAK1 GDA/BL	156918	0.0010053	** p<0.01
HSP90.1 BRI1 BL vs BRI1/BAK1 BL	29957	0.4136709	insignificant
HSP90.1 BRI1 BL vs HSP90.1/BRI1 GDA	70471	0.0010053	** p<0.01
HSP90.1 BRI1 BL vs BRI1/BAK1 GDA	21009	0.7893985	insignificant
HSP90.1 BRI1 BL vs HSP90.1/BRI1 GDA/BL	42706	0.0622925	insignificant
HSP90.1 BRI1 BL vs BRI1/BAK1 GDA/BL	142745	0.0010053	** p<0.01
BRI1/BAK1 BL vs HSP90.1/BRI1 GDA	44202	0.0468989	* p<0.05
BRI1/BAK1 BL vs BRI1/BAK1 GDA	0.9583	0.8999947	insignificant
BRI1/BAK1 BL vs HSP90.1/BRI1 GDA/BL	16872	0.8999947	insignificant

BRI1/BAK1 BL vs BRI1/BAK1 GDA/BL	116249	0.0010053	** p<0.01
HSP90.1/BRI1 GDA vs BRI1/BAK1 GDA	53309	0.0067400	** p<0.01
HSP90.1/BRI1 GDA vs HSP90.1/BRI1 GDA/BL	23792	0.6743169	insignificant
HSP90.1/BRI1 GDA vs BRI1/BAK1 GDA/BL	63007	0.0010053	** p<0.01
BRI1/BAK1 GDA vs HSP90.1/BRI1 GDA/BL	25339	0.6103739	insignificant
BRI1/BAK1 GDA vs BRI1/BAK1 GDA/BL	126917	0.0010053	** p<0.01
HSP90.1/BRI1 GDA/BL vs BRI1/BAK1 GDA/BL	85971	0.0010053	** p<0.01

Supplementary Table S15. Statistical analysis of Fig.S1C (Col-0).

treatments	Tukey HSD	Tukey HSD	Tukey HSD
pair	Q statistic	p-value	inferfence
0 nM BL vs 0,1 nM BL	53.299	0.0038617	** p<0.01
0 nM BL vs 1 nM BL	0.5576	0.8999947	insignificant
0 nM BL vs 10 nM BL	27.666	0.4476386	insignificant
0 nM BL vs 60 nM BL	56.223	0.0017905	** p<0.01
0 nM BL vs 100 nM BL	81.814	0.0010053	** p<0.01
0 nM BL vs 1000 nM BL	146.110	0.0010053	** p<0.01
0,1 nM vs 1 nM BL	40.370	0.0694273	insignificant
0,1 nM vs 10 nM BL	85.552	0.0010053	** p<0.01
0,1 nM vs 60 nM BL	109.325	0.0010053	** p<0.01
0,1 nM vs 100 nM BL	138.553	0.0010053	** p<0.01
0,1 nM vs 1000 nM BL	198.300	0.0010053	** p<0.01
1 nM BL vs 10 nM BL	29.721	0.3558119	insignificant
1 nM BL vs 60 nM BL	55.123	0.0024013	** p<0.01
1 nM BL vs 100 nM BL	77.297	0.0010053	** p<0.01
1 nM BL vs 1000 nM BL	136.311	0.0010053	** p<0.01
1 nM BL vs 60 nM BL	32.620	0.2450545	insignificant
10 nM BL vs 100 nM BL	58.617	0.0010053	** p<0.01
10 nM BL vs 1000 nM BL	127.817	0.0010053	** p<0.01
60 nM BL vs 100 nM BL	22.299	0.6725371	insignificant
60 nM BL vs 1000 nM BL	90.497	0.0010053	** p<0.01
100 nM BL vs 1000 nM BL	72.637	0.0010053	** p<0.01

Supplementary Table S16. Statistical analysis of Fig.S1C (*hsp90.1*).

treatments	Tukey HSD	Tukey HSD	Tukey HSD
pair	Q statistic	p-value	inferfence
0 nM BL vs 0,1 nM BL	104.843	0.0010053	** p<0.01
0 nM BL vs 1 nM BL	169.920	0.0010053	** p<0.01
0 nM BL vs 10 nM BL	189.566	0.0010053	** p<0.01
0 nM BL vs 60 nM BL	23.842	0.6088083	insignificant
0 nM BL vs 100 nM BL	43.170	0.0395542	* p<0.05
0 nM BL vs 1000 nM BL	19.500	0.7880550	insignificant
0,1 nM vs 1 nM BL	51.268	0.0062649	** p<0.01
0,1 nM vs 10 nM BL	66.064	0.0010053	** p<0.01
0,1 nM vs 60 nM BL	76.804	0.0010053	** p<0.01
0,1 nM vs 100 nM BL	66.333	0.0010053	** p<0.01
0,1 nM vs 1000 nM BL	119.487	0.0010053	** p<0.01
1 nM BL vs 10 nM BL	14.880	0.8999947	insignificant
1 nM BL vs 60 nM BL	134.871	0.0010053	** p<0.01
1 nM BL vs 100 nM BL	128.993	0.0010053	** p<0.01
1 nM BL vs 1000 nM BL	182.845	0.0010053	** p<0.01
1 nM BL vs 60 nM BL	151.980	0.0010053	** p<0.01
10 nM BL vs 100 nM BL	147.843	0.0010053	** p<0.01
10 nM BL vs 1000 nM BL	201.801	0.0010053	** p<0.01
60 nM BL vs 100 nM BL	16.214	0.8999947	insignificant
60 nM BL vs 1000 nM BL	41.390	0.0564738	insignificant
100 nM BL vs 1000 nM BL	61.229	0.0010053	** p<0.01

Supplementary Table S17. Statistical analysis of Fig.S1C (*hsp90.2*).

treatments	Tukey HSD	Tukey HSD	Tukey HSD
pair	Q statistic	p-value	inferfence
0 nM BL vs 0,1 nM BL	39.325	0.0845475	insignificant
0 nM BL vs 1 nM BL	111.452	0.0010053	** p<0.01
0 nM BL vs 10 nM BL	61.618	0.0010053	** p<0.01
0 nM BL vs 60 nM BL	13.484	0.8999947	insignificant
0 nM BL vs 100 nM BL	0.7472	0.8999947	insignificant
0 nM BL vs 1000 nM BL	63.815	0.0010053	** p<0.01
0,1 nM vs 1 nM BL	69.347	0.0010053	** p<0.01
0,1 nM vs 10 nM BL	31.157	0.2982228	insignificant
0,1 nM vs 60 nM BL	23.217	0.6347438	insignificant
0,1 nM vs 100 nM BL	32.902	0.2362003	insignificant
0,1 nM vs 1000 nM BL	100.124	0.0010053	** p<0.01
1 nM BL vs 10 nM BL	19.786	0.7761135	insignificant
1 nM BL vs 60 nM BL	88.742	0.0010053	** p<0.01
1 nM BL vs 100 nM BL	106.847	0.0010053	** p<0.01
1 nM BL vs 1000 nM BL	169.408	0.0010053	** p<0.01
1 nM BL vs 60 nM BL	48.325	0.0131526	* p<0.05
10 nM BL vs 100 nM BL	56.862	0.0015386	** p<0.01
10 nM BL vs 1000 nM BL	109.606	0.0010053	** p<0.01
60 nM BL vs 100 nM BL	0.6846	0.8999947	insignificant
60 nM BL vs 1000 nM BL	72.701	0.0010053	** p<0.01
100 nM BL vs 1000 nM BL	72.129	0.0010053	** p<0.01

Supplementary Table S18. Statistical analysis of Fig.S5B.

treatments<br>pair	Tukey HSD<br>Q statistic	Tukey HSD<br>p-value	Tukey HSD<br>inferfence
WS-2 vs WS-2 BL	110.942	0.0010053	** p<0.01
WS-2 vs WS-2 GDA	133.533	0.0010053	** p<0.01
WS-2 vs *bri1-4*	297.232	0.0010053	** p<0.01
WS-2 vs *bak1-1*	108.030	0.0010053	** p<0.01
WS-2 BL vs WS-2 GDA	215.470	0.0010053	** p<0.01
WS-2 BL vs *bri1-4*	379.168	0.0010053	** p<0.01
WS-2 BL vs *bak1-1*	210.743	0.0010053	** p<0.01
WS-2 GDA vs *bri1-4*	135.731	0.0010053	** p<0.01
WS-2 GDA vs *bak1-1*	45.339	0.0290713	* p<0.05
bri1-4 vs *bak1-1*	202.068	0.0010053	** p<0.01

Supplementary Table S19. Statistical analysis of Fig.S5D.

	treatments pair	Tukey HSD Q statistic	Tukey HSD p-value	Tukey HSD inferfence
Col-0	control vs BL	133.751	0.0010053	** p<0.01
	control vs GDA	0.2810	0.8999947	insignificant
	BL vs GDA	104.168	0.0010053	** p<0.01
hsp90.1	control vs BL	0.6771	0.8772375	insignificant
	control vs GDA	33.958	0.0701677	insignificant
	BL vs GDA	43.682	0.0182320	* p<0.05
hsp90.2	control vs BL	77.235	0.0010053	** p<0.01
	control vs GDA	82.263	0.0010053	** p<0.01
	BL vs GDA	0.8133	0.8230646	insignificant

Supplementary Table S20. Statistical analysis of Fig.S5E.

		p-values from t-test
Col	control	
	BL	0.0010053
hsp90.1	control	0,05156282
	BL	0,00230128
hsp90.2	control	0,14868104
	BL	0,71938764

Supplementary Table S21. Statistical analysis of Fig.S5F.

		p-values from t-test
Col	control	
	BL	0.0010053
hsp90.1	control	2,10407E-05
	BL	0,005081537
hsp90.2	control	0,000488143
	BL	0,00949886

Supplementary Table S22. Statistical analysis of Fig.S5G.

		p-values from t-test
Col	control	
	BL	0.0010053
hsp90.1	control	0,00022173
	BL	0,00571891
hsp90.2	control	0,05744368
	BL	0,03853117

Supplementary Table S23. Statistical analysis of Fig.S6I.

treatments	Tukey HSD	Tukey HSD	Tukey HSD
pair	Q statistic	p-value	inferfence
control (BL-) vs GDA	226.408	0.0010053	** p<0.01
control (BL-) vs control (BL+)	127.426	0.0010053	** p<0.01
control (BL-) vs GDA (BL+)	78.173	0.0010053	** p<0.01
GDA vs control (BL+)	61.371	0.0010053	** p<0.01
GDA vs GDA (BL+)	186.577	0.0010053	** p<0.01
control (BL+) vs GDA (BL+)	75.638	0.0010053	** p<0.01

Supplementary Table S24. Statistical analysis of Fig.S6J.

treatments	Tukey HSD	Tukey HSD	Tukey HSD
pair	Q statistic	p-value	inferfence
WT (BL-) vs WT (BL+)	104.859	0.0010053	** p<0.01
WT (BL-) vs *hsp90.1* (BL-)	111.758	0.0010053	** p<0.01
WT (BL-) vs *hsp90.1* (BL+)	99.545	0.0010053	** p<0.01
WT (BL-) vs *hsp90.2* (BL-)	42.236	0.0344292	* p<0.05
WT (BL-) vs *hsp90.2* (BL+)	131.776	0.0010053	** p<0.01
WT (BL+) vs *hsp90.1* (BL-)	0.1264	0.8999947	insignificant
WT (BL+) vs *hsp90.1* (BL+)	15.118	0.8912025	insignificant
WT (BL+) vs *hsp90.2* (BL-)	150.709	0.0010053	** p<0.01
WT (BL+) vs *hsp90.2* (BL+)	14.387	0.8999947	insignificant
hsp90.1 (BL-) vs *hsp90.1* (BL+)	17.317	0.8015711	insignificant
hsp90.1 (BL-) vs *hsp90.2* (BL-)	161.518	0.0010053	** p<0.01
hsp90.1 (BL-) vs *hsp90.2* (BL+)	13.766	0.8999947	insignificant
hsp90.1 (BL+) vs *hsp90.2* (BL-)	151.833	0.0010053	** p<0.01
hsp90.1 (BL+) vs *hsp90.2* (BL+)	32.708	0.1898402	insignificant
hsp90.2 (BL-) vs *hsp90.2* (BL+)	187.027	0.0010053	** p<0.01

Supplementary Table S25. Statistical analysis of Fig.S7E.

Treatments	Tukey HSD Q statistic	Tukey HSD p-value	Tukey HSD inferfence
BAK1 control vs BAK1 GDA	187025	0.0010053	** p<0.01

Supplementary Table S26. Statistical analysis of Fig.S7F.

Treatments	Tukey HSD Q statistic	Tukey HSD p-value	Tukey HSD inferfence
BAK1 control vs BAK1 GDA	44.485	0.0156769	* p<0.05

Supplementary Table S27. Statistical analysis of Fig.S8E.

treatments	Tukey HSD<br><br>Q statistic	Tukey HSD<br><br>p-value	Tukey HSD<br><br>inferfence
HSP90.1/BRI1 vs HSP90.2/BRI1	53953	0.0019276	** p<0.01
HSP90.1/BRI1 vs BRI1/BAK1	82904	0.0010053	** p<0.01
HSP90.2/BRI1 vs BRI1/BAK1	32969	0.0677473	insignificant

Supplementary Table S28. Statistical analysis of Fig.S8F.

treatments	Tukey HSD Q statistic	Tukey HSD p-value	Tukey HSD inferfence
HSP90.1/BRI1 vs HSP90.2/BRI1	78048	0.0010053	** p<0.01
HSP90.1/BRI1 vs BRI1/BAK1	90797	0.0010053	** p<0.01
HSP90.2/BRI1 vs BRI1/BAK1	16381	0.4878172	insignificant
